# Low-Light Image and Video Enhancement for More Robust Computer Vision Tasks: A Review

**DOI:** 10.3390/jimaging11040125

**Published:** 2025-04-21

**Authors:** Mpilo M. Tatana, Mohohlo S. Tsoeu, Rito C. Maswanganyi

**Affiliations:** 1 Department of Electronic and Computer Engineering, Durban University of Technology, Durban 4001, South Africa; 21959577@dut4life.ac.za; 2 Steve Biko Campus, Durban University of Technology, Durban 4001, South Africa; mohohlot@dut.ac.za

**Keywords:** light enhancement, video de-flickering, zero-shot learning, action recognition, object detection, computer vision, criminal activity

## Abstract

Computer vision aims to enable machines to understand the visual world. Computer vision encompasses numerous tasks, namely action recognition, object detection and image classification. Much research has been focused on solving these tasks, but one that remains relatively uncharted is light enhancement (LE). Low-light enhancement (LLE) is crucial as computer vision tasks fail in the absence of sufficient lighting, having to rely on the addition of peripherals such as sensors. This review paper will shed light on this (focusing on video enhancement) subfield of computer vision, along with the other forementioned computer vision tasks. The review analyzes both traditional and deep learning-based enhancers and provides a comparative analysis on recent models in the field. The review also analyzes how popular computer vision tasks are improved and made more robust when coupled with light enhancement algorithms. Results show that deep learners outperform traditional enhancers, with supervised learners obtaining the best results followed by zero-shot learners, while computer vision tasks are improved with light enhancement coupling. The review concludes by highlighting major findings such as that although supervised learners obtain the best results, due to a lack of real-world data and robustness to new data, a shift to zero-shot learners is required.

## 1. Introduction

Light enhancement (LE) networks aim to enhance low-light and poorly lit visual data, to allow more information to be extracted from the visual data for further processing. The simple brightening of an image does not lead to desired results. Firstly, in low-light conditions, not all objects of the image are equally deprived of lighting; thus, the enhancement of objects on an image needs to be performed with consideration of local luminosity. Secondly, images captured in low-lighting conditions often incur noise; thus, a major challenge exists in enhancing these lighting conditions without enhancing the unwanted noise; therefore, light enhancement is a non-trivial task. LE methods can be divided into two groups, traditional learning and deep learning. These are further sub-divided into histogram equalization, Retinex, Dehazing and statistical techniques for traditional learning, and supervised learning, unsupervised learning, semi-supervised learning and zero-shot learning for deep learning [1]. Traditional learning methods like those employed in [2,3,4,5] were commonly used before the rise of deep learning. Some, like histogram equalization, are still widely employed alongside deep learning methods due to their abilities to better handle specific tasks; in the case of histogram equalization, that being maintaining contrast amongst neighboring pixels and enhancing the dynamic range of an image. Deep learning methods have gained popularity in the LE domain due to the improvements in neural networks over the years that have made them more accurate and faster. Although deep learning methods exhibit the forementioned crucial benefits, one major drawback is their need for large real-world and diverse training data.

Within the domain of LE, image enhancement enjoys the bulk of most research over video enhancement. Algorithms that aim to enhance videos simply extend image enhancement networks to videos by handling the videos frame by frame. This causes temporal inconsistencies in the processed video, which leads to various artefacts such as blurs, poor color grading and flickering. Deblurring is a technique that aims to recover sharp images from blurry ones. When an image is blurred, mathematically, it is convoluted with a blurring kernel (also termed Point Spread Function, “PSF”) resulting in the blurred image. This blur can be due to object or camera shake, out-of-focus objects, or slow shutter speed of camera to name a few. Deblurring may be categorized as being uniform or non-uniform, as well as blind or non-blind. Uniform blur means that the kernel that caused the blur throughout the frame is constant; the opposite is true with non-uniform blur (also termed local blur). Blind blur occurs when the kernel that caused the blur effect is unknown, making it harder to deblur the image, while with non-blind blur, the blur-causing kernel is known. Real-world scenarios of blur are often blind and non-uniform. Deblurring networks have the following basic sequential layers: Convolutional layer, Recurrent Layer, Residual layer, Dense layer and Attention layer. The most common architectures include Deep Auto-Encoders, Generative Adversarial Networks (GANs), Cascaded Networks, Multi-scale Networks and Reblurring Networks.

Poor color grading (poor color transfer) occurs when an image or video that is in greyscale or is poorly colored (such as in the case of low-light images or videos) is poorly adjusted in color and tonal balance by an algorithm. This poor colorization manifests itself as either chromatic flickering or incorrect colorization of objects in the processed visual data along with a loss of color contrast. Flickering in processed videos is often caused by poor processing techniques that destroy the videos’ temporal coherence between neighboring video frames, causing artefacts in the processed video. To remove video flickers, exploitations of “the temporal information between neighboring frames” [6] is required. To ensure temporal information is maintained frame to frame, methods include using 3D Convolutional Networks (ConvNets) which capture both spatial and temporal information, along with LSTM or GRUs. Consideration is required, as while 3D ConvNets can capture both temporal and spatial information, this is only for a short period of time as compared to LSTM and GRUs, which can capture longer sequences but struggle at capturing spatial information compared to ConvNets.

Action recognition (AR) is the ability of machines to recognize human activities in videos. “Actions are ‘meaningful interactions’ between humans and the environment” [7]. Actions can be short term or long term, and in the case of long-term actions, recurrence is required to ensure that the network is able to “remember” actions that happened at the start of the sequence. Recurrence also assists the model in understanding the sequence in which actions occur. Due to this, many action recognition models leverage some sort of recurrent networks, be it LSTMs or GRUs.

The methodologies chosen in this review were chosen for being the latest popular models, or being popular models whose robustness and effectiveness has stood the test of time. Examples of such models would be recent and popular models like Zero DCE (proposed in 2020) [8] and much older models like the YOLO algorithms; YOLOv1 [9] was proposed in 2015, but due to its faster than real-time performance, it continues to be utilized for many real-time detection tools till this day.

The contributions of this review are as follows:

1. Provides a survey of recent and most impactful LE models, video artefacts removal networks, action recognition and object detection algorithms. By so doing, this review provides a framework which researchers can use to build nighttime-enabled computer vision algorithms such as autonomous criminal activity detectors, where similar algorithms have been attempted using traditional methods, with little success. This review, on the other hand, studies deep learning methods which have been proven to outperform traditional learning methods.
2. Recent and popular computer vision models are compared with each other, both qualitatively and quantitatively, providing a holistic evaluation of each model.
3. The survey summarizes the most popular datasets utilized in the various networks mentioned, while also giving an overview of the data format of these datasets and related information. The review also gives a methodology on how the datasets can be manipulated to be more representative of real-world data, which leads to improved model performances.
4. Finally, this review lays out challenges that face the highlighted computer vision tasks, and a means for tackling these challenges.

The rest of this review is organized as follows:

Section 2 discusses different models in LE and the various learning techniques used. Artefact removal is reviewed in Section 3, while artefact removal coupled with light enhancement is analyzed in Section 4. Section 5 delves into action recognition and object detection, and Section 6 focuses on how action detection and object detection can be improved in low-light conditions when coupled with LE models. Section 7 talks about the various datasets used for training and testing LE models, while Section 8 paints a picture of the challenges that remain to be tackled in LE. Section 9 concludes the findings made in this paper.

## 2. LE Techniques

**Figure 1:**
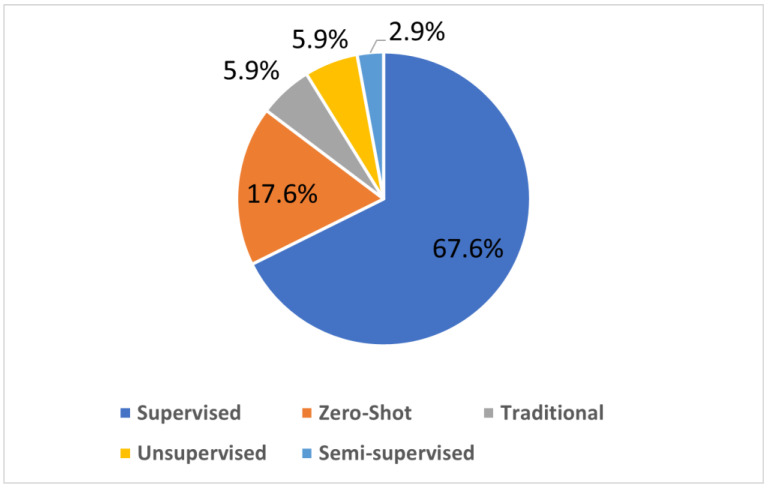
Popularity of various LE learning strategies [1].

Light enhancement methods can be divided into two main categories, namely traditional learning and deep learning. Most enhancers employ deep learning techniques as a result of their superior processing speeds and accuracy; therefore, this paper will focus on deep learning-based enhancers. The enhancers discussed will be classified into four learning strategies, supervised, zero-shot, traditional unsupervised and semi-supervised. The popularity of these learning strategies is visualized in Figure 1.

### 2.1. Traditional Learning-Based Enhancers

Traditional learning-based enhancers make use of techniques such as histogram equalization statistical techniques, gray level transformation and Retinex theory. Traditional enhancers require less training data while also using less computational resources.

**Figure 2:**
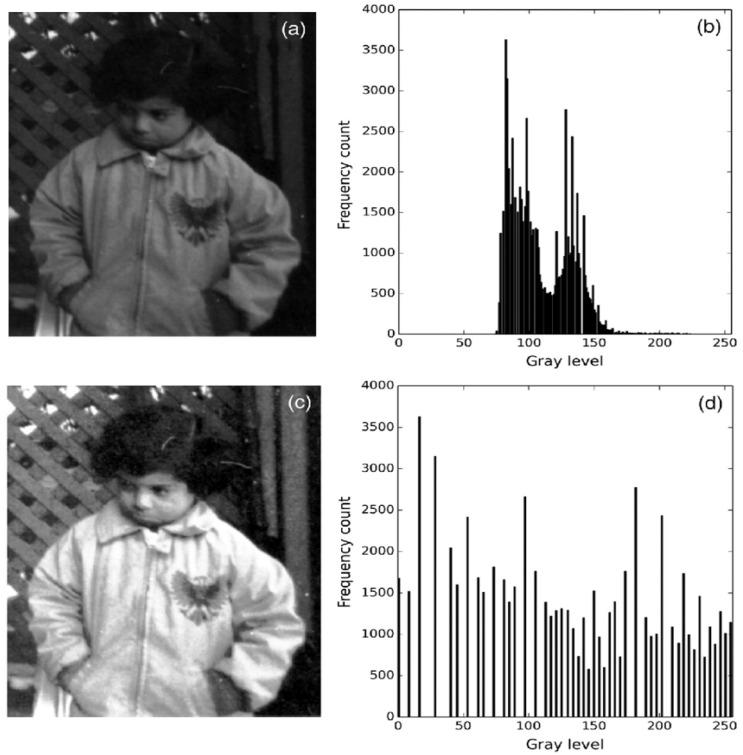
Histogram equalization illustration [13]. (**b**) The histogram of (**a**), which shows a highly condensed histogram. (**d**) The histogram of (**c**), which shows the equalized version of the histogram in (**b**). (Reprinted from [13]).

Histogram equalization (HE) techniques such as those employed in [10,11,12] are used to improve the contrast of the image. This is achieved by spreading the pixel intensity throughout the image, pictorially demonstrated in Figure 2 [13].

The other popular traditional learning technique for light enhancement, Retinex theory, takes inspiration from the biology of the human eye and was first introduced in [3]. The human eye is able to detect object color under changing illumination conditions, and Retinex theory aims to imitate this. Retinex theory decomposes an image into two parts, illumination and reflectance. The reflectance mapping represents the colors of objects in the image, while illumination represents the intensity of light in the image. To enhance the image, the reflectance is enhanced while ensuring that illumination is adjusted such that the image is bright enough to perceive objects in the image (not too dim, not too bright). Some enhancers that have employed Retinex theory in recent years have been discussed in [14,15,16].

**Figure 3:**
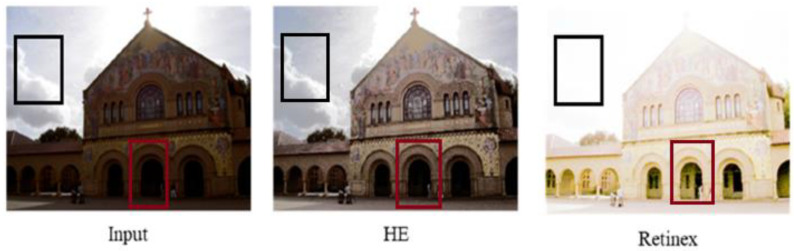
Pitfalls of traditional learning enhancers [15]. The input image requires enhancement, specifically the building, while preserving (minimal enhancement) the contrasts of the clouds. The histogram-equalized image can slightly enhance parts of the building while preserving the contrast in the clouds, seen in the black square, but fails to enhance the darker parts of the image highlighted in the red square. The Retinex-enhanced image over-enhances the image and as a result, contrast in the clouds (black square) is lost even though the darker parts of the building are now visible. The Retinex-enhanced image also inadvertently enhances noise as well. (Reprinted from [15]).

The pitfalls of these traditional methods lie in that these methods are limited in their ability to handle enhancing images with complex lighting conditions, such as back-lit images, front-lit images, and any images where the lighting conditions are not uniform. Traditional enhancers treat an image as though the poor lighting conditions are equally shared by all pixels in the image and thus often apply global transformation techniques. This is seen in Figure 3, which illustrates the major issue with traditional learners. The input image is back-lit, with the sky and clouds in the background clearly visible and thus requiring very little if any enhancement, while the cathedral is not well lit and thus requires enhancement. HE is able to enhance the cathedral walls, and maintain some resemblance of the sky to the original image, but in doing so sacrifices the ability to enhance darker regions, noted in red. Retinex is able to enhance these darker regions that HE failed to enhance, but in doing so, over-enhances the already well-lit parts of the images; contrast is lost in these parts, making it harder to distinguish the start and end points of various objects. Adaptive variants of traditional enhancers exist that consider the local differences in an image [17]. These variants such as Contrast-Limited Adaptive Histogram Equalization (CLAHE), a variant of HE, are better at capturing local contrasts and preserving edges, but in doing so sacrifice runtime for performance. Although the overall performance of the algorithm is improved by said variations, it still falls behind deep learners, as they (traditional learners) are not very robust (poor ability to adapt to a wide range of lighting conditions), poor at detail preservation and apply basic techniques to noise reduction, which often results in noise being amplified in the final image along with the desired signal.

### 2.2. Supervised Learning-Based Enhancers

Supervised learning in LE requires labeled and paired data. The data used are of the same scene both in low-light and optimal lighting conditions. For this reason, supervised learning-based models often suffer from a lack of a large diverse dataset, often leading to the use of synthetic data, which fail to capture the natural variations in the lighting in a scene (i.e., naturally in a scene, often some objects may appear dark while others appear over-illuminated). Even with the mammoth task of requiring diverse and paired datasets of the same scene, supervised enhancers continue to dominate in terms of choice, due to them continually outperforming other enhancers on benchmark tests.

**Figure 4:**
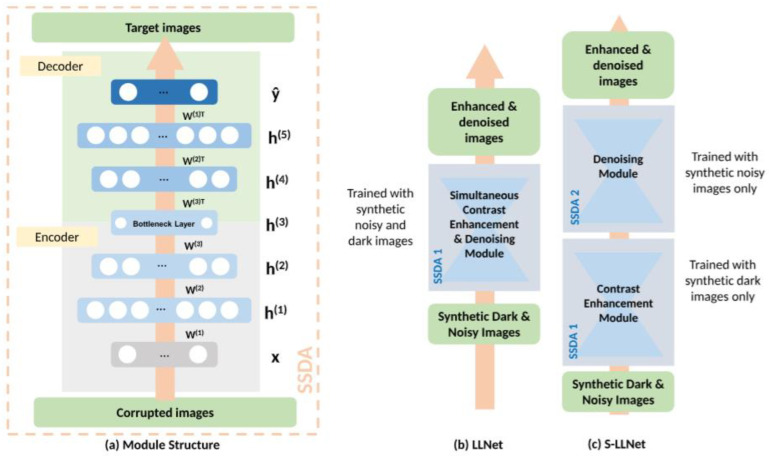
LLNet architecture. In (**a**), x and $\hat{y}$ are the corrupted and reconstructed images, respectively, and h^(i)^ are the hidden layers [18]. (**b**) The simultaneous enhancement and denoising LLNet. (**c**) LLNet with sequential enhancement followed by denoising. (Reprinted with permission from [18] 2017, Elsevier, Amsterdam, The Netherlands).

One of the first supervised LE models employed deep encoders to adaptively enhance and denoise images. LLNet [18] enhances contrast such that the image improvements are completed relative to local neighbors. This helps prevent the model from enhancing already bright regions, which is a challenge that plagues many enhancers. The network is also trained to recognize stable features of images even in the presence of noise, to equip the model with denoising capabilities. LLNet takes its inspiration from Sparse Stacked Denoising Autoencoders (SSDAs) [19] and their denoising capabilities. The SSDA is derived from research performed by [19] (illustrated in Figure 4a), which showed that a model is able to find better parameter space during back-propagation by stacking denoising autoencoders (DAs). Let y**_i_** be the uncorrupted desired image and x**_i_** the corrupted input version of y**_i_**, where i is an element of positive integers; DA is thus defined as follows:

(1) $$h\left(x_{i}\right)=\sigma (Wx_{i}+b)$$

(2) $$\hat{y}\left(x_{i}\right)=\sigma (W^{\prime }h\left(x_{i}\right)+b^{\prime })$$

where ${\sigma \left(x\right)=\left(1+\exp\left(-x\right)\right)}^{-1}$ is the element-wise sigmoid activation function, the hidden layers defined by $h_{i}$, $y_{i}$ are approximated by $\hat{y}\left(x_{i}\right)$. The weights and biases are defined as Θ=[ W, b, W′, b′ ].

The authors developed two models, vanilla LLNet, which simultaneously enhances and denoises and the staged LLNet, which first enhances and then denoises, as illustrated in Figure 4b,c respectively. The results in the comparisons of the two LLNet models show that vanilla LLNet outperforms staged LLNet on numerous metrics, which supports the idea that simultaneous enhancing and denoising yields more desirable results than sequential enhancing and denoising. This is a key observation as most low-light visual data are consumed by noise, and enhancers have the unintended tendency of enhancing said noise. This observation is also supported by coupled enhancers discussed later in this paper.

Table 1 shows a performance comparison between LLNet and some non-deep learning techniques on synthetic and real dark images. Table 2 compares LLNet with the same traditional learning strategies but with dark and noisy synthetic data. In both cases, both the vanilla LLNet and S-LLNet outperform the traditional LE techniques (with LLNet outperforming S-LLNet) while histogram equalization performs the worst. The various models are evaluated using the Peak Signal to Noise Ratio (PSNR) and Structural Similarity Index Measure (SSIM). PSNR is given by (3) and (4), where n in PSNR is the number of bits per pixel, generally eight bits, and MSE is the mean square error. The Structural Similarity Index Measure (SSIM), first formulated by [20], per pixel is formulated in (5). The SSIM is used to explore structural information in an image. The structures as defined by [20] are “those attributes that represent the structure of objects in the scene, independent of the average luminance and contrast” [20]. In (5), x and y are the inputs from the unprocessed and processed images, respectively. l(x,y) defines the luminance component, the contrast component is defined by c(x,y), and the structure component by s(x,y). These components are weighted by the exponents a,b,c, respectively.

(3) $$PSNR=10{log}_{10}(\frac{{\left(2^{n}-1\right)}^{2}}{MSE})$$

(4) $$MSE=\frac{1}{mn}\sum_{i=0}^{m-1}\sum_{j=0}^{n-1}{\left[I\left(i,j\right)-K(i,j)\right]}^{2}$$

(5) $$SSIM(x,y)=(l(x,y){)}^{\alpha }(c(x,y){)}^{\beta }(s(x,y){)}^{\gamma }$$

**Figure 5:**
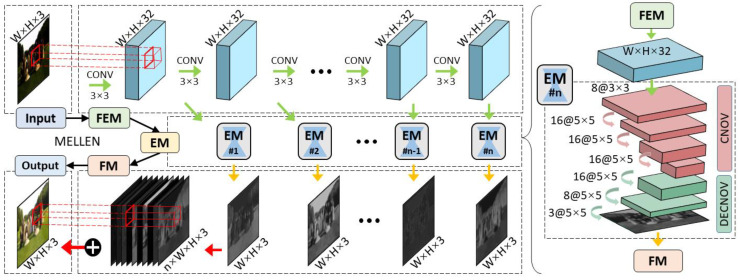
MBLLEN Model pipeline [21]. (Reprinted from [21].)

Lv et al. [21] proposed a multi-branch low-light enhancement network (MBLLEN) to extract features from different levels and apply enhancement via multiple subnets. The branches proposed are the feature extraction module (FEM), which extracts image features and feeds the output to the Enhancement Module (EM). The EM enhances images and the outputs from the EM are concatenated in the Fusion Module (FM) via multi-branch fusion, as illustrated in Figure 5.

For training and testing, the model utilized a synthesized version of the VOC Dataset [22] (Poisson noise was added to images). The model also employed the e-Lab Video Data Set (e-VDS) [23] for training and testing its modified low-light video enhancement (LLVE) network. Both datasets were altered with random gamma adjustment to synthesize low-light data. This process of creating synthetic low-light data means that the model is poorly suited for real-world scenarios, which was observed in its poor performance in extremely low-light videos, resulting in flickering in the videos processed [6]. The model also did not employ color correcting error metrics, which caused the model’s color inconsistencies in the processed videos. Another limitation of the model is its 3 s runtime, making it unsuitable for real-world applications. Table 3a–c show the self-reported results from different evaluations of the MBLLEN algorithm. Table 3a,b show the comparison of MBLLEN on dark images and dark + noisy images, respectively. Table 3c shows the video enhancement version of the model, which utilizes 3D convolutions instead of 2D convolutions. VIF [24] is the Visual Information Fidelity used to determine if image quality after embedding has improved, formulated in (6)–(8). In (6), the numerator determines the mutual information between the reference image (${\vec{C}}^{N,j}$) and corrupted image (${\vec{F}}^{N,j}$) given subband statistics ($s^{N,j}$).

(6) $$\begin{array}{c} VIF=\frac{\sum_{j\in \;\mathrm{subbands}\;I}\;\left({\vec{C}}^{N,j};{\vec{F}}^{N,j}|s^{N,j}\right)}{\sum_{j\in \;\mathrm{subbands}\;I}\;I\left({\vec{C}}^{N,j};{\vec{E}}^{N,j}|s^{N,j}\right)} \end{array}$$

(7) $$\begin{array}{c} I\left({\vec{C}}^{N};{\vec{F}}^{N}|s^{N}\right)=\frac{1}{2}\sum_{x=1}^{W}\;\sum_{y=1}^{H}\;{log}_{2}\left(1+\frac{g_{x}^{2}s_{x}^{2}\lambda_{y}}{\sigma_{v}^{2}+\sigma_{n}^{2}}\right) \end{array}$$

(8) $$\begin{array}{c} I\left({\vec{C}}^{N};{\vec{E}}^{N}|s^{N}\right)=\frac{1}{2}\sum_{x=1}^{W}\;\sum_{y=1}^{H}\;{log}_{2}\left(1+\frac{s_{x}^{2}\lambda_{y}}{\sigma_{n}^{2}}\right) \end{array}$$

In (7) and (8):

- ${\vec{C}}^{N}$: Reference image;
- ${\vec{F}}^{N}$: Distorted image;
- $g_{x,y}$: Gain;
- $s_{x,y}$: variance in reference subband coefficients;
- $\lambda_{y}$: variance in the wavelet coefficients for spatial location y;
- $\sigma_{v}$: variance in visual noise;
- $\sigma_{n}$: variance in additive distortion noise.

The Lightness Order Error (LOE) [25] is used to measure the distortion of lightness in enhanced images. RD(x) is the relative order difference of the lightness between the original image P and its enhanced version P′ for pixel x, which is defined by (10). The pixel number is defined by m, and the lightness component of the pixel x before and after enhancement is defined by L(x) and L′(x).

(9) $$LOE=\frac{1}{M}\sum_{x=1}^{m}RD(x)$$

(10) $$RD(x)=\sum_{y=1}^{m}\;U(L(x),L(y))⊕U\left(L^{\prime }(x),L^{\prime }(y)\right)$$

TMQI [26] defines the Tone-Mapped Image Quality Index, which combines a multi-scale structural fidelity measure and a statistical naturalness measure to assess the quality of tone-mapped images. In (11), the TMQI is defined as Q, a ∈[0, 1] adjusts the relative importance of the two components (structural fidelity and statistical naturalness), and α and β calculate their sensitivity.

(11) $$Q=aS^{a}+\left(1-a\right)N^{\beta }$$

**Table 3:** (**a**). Qualitive comparison of MBLLEN with other LLIE networks on dark image only (Reprinted from [21].) (**b**) Qualitive comparison of MBLLEN with other LLIE networks, using dark and noisy images. (Reprinted from [21].) (**c**) Qualitative comparison of MBLLEN and MBLLVEN with other enhancers on low-light video enhancement. The best-performing model is highlighted in bold. (↑) Indicates higher values are desirable, the opposite is true (Reprinted from [21].)

(**a**)					
	**PSNR (↑)**	**SSIM (↑)**	**VIF (↑)**	**LOE (↓)**	**TMQI (↑)**
Input	12.80	0.43	0.38	606.85	0.79
SRIE [27]	15.84	0.59	0.43	788.53	0.82
BPDHE [28]	15.01	0.59	0.39	607.43	0.81
LIME [28]	15.16	0.60	0.44	1215.58	0.82
MF [29]	18.48	0.67	0.45	882.24	0.84
Dong [30]	17.80	0.64	0.37	1398.35	0.82
NPE [31]	17.65	0.68	0.43	1051.15	0.84
DHECI [32]	18.18	0.68	0.43	606.98	0.87
WAHE [33]	17.64	0.67	0.48	648.29	0.84
Ying [5]	19.66	0.73	0.47	892.56	0.86
BIMEF [25]	19.80	0.74	0.48	675.15	0.85
MBLLEN	**26.56**	**0.89**	**0.55**	**478.02**	**0.91**
(**b**)					
	**PSNR**	**SSIM**	**VIF**	**LOE**	**TMQI**
WAHE	17.91	0.62	0.40	771.34	0.83
MF	19.37	0.67	0.39	896.67	0.84
DHECI	18.03	0.67	0.36	687.60	0.86
Ying	18.61	0.70	0.40	928.13	0.86
BIMEF	20.27	0.73	0.41	725.72	0.85
MBLLEN	**25.97**	**0.87**	**0.49**	**573.14**	**0.90**
(**c**)					
	**LIME**	**Ying**	**BIMEF**	**MBLLEN**	**MBLLVEN**
PSNR	14.26	22.36	19.80	19.71	**24.98**
SSIM	0.59	0.78	0.76	**0.88**	0.83

KinD and KinD++ [34] take inspiration from Retinex theory [35] and propose the decomposition of an input image into two components, the illumination map for light adjustments and reflectance for degradation removal. The KinD network architecture can also be divided into the Layer Decomposition Network, Reflectance Restoration Network and Illumination Adjustment Network.

**Layer Decomposition Net:** The layer is responsible for the decomposition of the image into its components, the reflectance and illumination maps. A problem exists as there exists no ground-truth for these mappings. The layer overcomes this through the use of loss functions and images of varying lighting configurations. To enforce reflectance, the model utilizes (12), where the reflectance of two paired images is given by ${(R}_{L},\;R_{H})$. In (12), L denotes the loss of the reflectance similarity (rs) in the layer decomposition (LD); hence, we denote this loss as $L_{rs}^{LD}$. Similar notation is applied for (13) up till (18). For (12) up to (18), I denotes the image, R the reflectance map and L the illumination maps with subscripts to emphasize the difference in lighting of the images. The ℓ^1^ norm is represented by ||◦||_1_.

(12) $$L_{rs}^{LD}=|{\left|R_{L}-R_{H}\right||}_{1}$$

To ensure that the illumination maps (L_L_, L_H_) of paired images are piecewise smooth and mutually consistent, (13) is used to enforce this. In (13), the image gradients are represented by ∇ and a small epsilon (ε) is added to prevent division by zero.

(13) $$L_{is}^{LD}=\frac{{\left|\left|\nabla L_{L}\right|\right|}_{1}}{max(\left|\nabla I_{L}\right|,\epsilon )}+\frac{{\left|\left|\nabla L_{H}\right|\right|}_{1}}{max(\left|\nabla I_{H}\right|,\epsilon )}$$

Mutual consistency is enforced (14) to ensure that strong edges are aligned, while weak ones are suppressed.

(14) $$L_{mc}^{LD}={\left|\left|M\cdot e^{-cM}\right|\right|}_{1},\;M=\left|{\nabla L}_{L}\right|+|{\nabla L}_{H}|\;$$

For reconstruction of the original image, the illumination and reflectance layers are recombined, and to ensure proper reconstruction, the reconstruction consistency is enforced by (15) and thus the total loss function is defined by (16).

(15) $$L_{rec}^{LD}=|{\left|I_{L}-R_{L}\cdot L_{L}\right||}_{1}+|{\left|I_{H}-R_{H}\cdot L_{H}\right||}_{1}$$

(16) $$L^{LD}=L_{rec}^{LD}+0.01L_{rs}^{LD}+0.08L_{is}^{LD}+0.1L_{mc}^{LD}$$

**Reflectance Restoration Net:** Brighter images are usually less degraded than darker ones. The reflectance restoration net takes advantage of this observation and uses the reflectance mappings of the brighter images as references. To restore the degraded reflectance (R), the module employs (17), where the restored reflectance is denoted by $\hat{R}$, and $R_{H}$ denotes the reference reflectance from brighter images.

(17) $$L_{RR}={\left|\left|\hat{R}-R_{H}\right|\right|}_{2}^{2}-SSIM\left(\hat{R},\;R_{H}\right){+|\left|\nabla \hat{R}-\nabla R_{H}\right||}_{2}^{2}$$

**Illumination Adjustment Net:** The illumination adjustment net employs paired illumination maps and a scaling factor (α) to adjust illumination while preserving edges and the naturalness of an image. Adjusting the illumination to ensure similarity between the target (L_t_) and manipulated illumination ($\hat{L})$ is guided by the loss function in (18).

(18) $$L_{IA}{=|\left|\hat{L}-L_{t}\right||}_{2}^{2}+|{\left|\left|\nabla \hat{L}\right|-\left|\nabla L_{t}\right|\right||}_{2}^{2}$$

### 2.3. Zero-Shot Learning-Based Enhancers

Supervised methods of learning require paired and labeled data of the same scene (dark and light), which are often hard to acquire and often lead to the use of synthetic datasets, where darkness is artificially created. ExCNet [36] and Zero-DCE [8] pioneered a new paradigm in light enhancement, zero-reference learning. Zero-reference learning derives its name from the fact that the training data are unpaired and unlabeled; rather, the model relies on carefully selected non-reference loss functions and parameters to achieve the desired results. These LLIE methods make use of light enhancement curves which dictate the output-enhanced pixel value for a given dark input pixel value.

One of the earliest adopters of zero-shot learning-based light enhancement, ExCNet (Exposure Correction Network) [36], used S-curve estimation to enhance back-lit images. The model’s greatest advantage over models of its time is its zero-shot learning strategy, which aims to enable the model to recognize and classify classes which it had not seen during training, simply by using prior knowledge and semantics. The authors designed a block-based loss function which maximizes the visibility of global features while maintaining local relative differences between the features. To reduce flickers and computational costs when processing videos, the model takes advantage of the parameters from previous frames in order to guide the enhancement of the next frame.

The S-curve comprises Ø_s_ and Ø_h_, which are the shadow and highlight parameters used to parameterize the curve, respectively. The shadow and highlight parameters assist in adjusting underexposed and overexposed regions, respectively. The curve is represented in (19), where x and $f\left(x:\phi_{s},\phi_{h}\right)$ are the input and output luminance values, respectively. The incremental function is represented as $f_{\Delta }\left(t\right)$ ∈ [0, 0.5].

The aim of ExCNet is to find the optimal parameter pair [$\phi_{s},\phi_{h}$] that restores the back-lit image *I*. The model goes through two stages, the luminance channel *I_l_* adjustment using intermediate S-curves and loss derivation (20), where *E*_i_ is the unary data term, *E_ij_* is the pairwise term and (λ) is a predefined constant.

The model’s greatest challenge is that it has a runtime of 23.28 s, which makes it a very poor candidate for real-time applications, along with its niche domain (only works for back-lit images).

(19) $$f\left(x:\phi_{s},\phi_{h}\right)=x+\phi_{s}\times f_{\Delta }(x)-\phi_{h}\times f_{\Delta }(1-x)$$

(20) $$L=\sum_{i}(E_{i}+\lambda \sum_{I\epsilon \Omega (i)}E_{ij})$$

**Figure 6:**
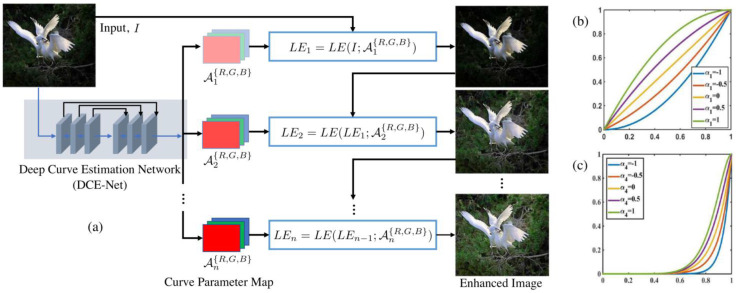
Zero DCE pipeline. (Reprinted from [37]). (**a**) Is the DCE_Net which estimates the enhancement curves used to iteratively enhance the low-light input image. In (**b**,**c**) the light enhancement curves are shown, where the input and output pixel values are represented in the x-axis and y-axis respectively. In (**b**) the number of iterations is 1 for all the curves with the adjustment parameter α changing from [−1,1] inclusive with a step of 0.5. In (**c**) the number of iterations for all the curves is 4, with the adjustment parameter changing as in (**b**).

Zero DCE [37] and its successor Zero DCE++ [38] are popular zero-shot low-light enhancement models, which use LE curves to estimate the best curve for low-light enhancement (LLE). These curves are aimed at achieving three goals:

- To avoid information loss, each enhanced pixel should be normalized within a value range of [0–1].
- For the sake of contrast preservation amongst neighboring pixels, the curves must be unvarying.
- The curves must be basic, and differentiable during back-propagation.
- These goals are achieved through (21) [37]
  (21) $$LE\left(I\left(x\right);a\right)=I\left(x\right)+aI(x)(1-I\left(x\right))$$

where x represents pixel coordinates, the input is denoted by $I\left(x\right)$, whose enhanced output is $\mathrm{LE}\left(I\left(x\right);a\right)$ and −1 < a<1 [37]. As seen in Figure 6, the model repeatedly enhances an image, and the enhancement occurs on each color channel (RGB) rather than on the entire image. Figure 6 also shows the Deep Curve Estimation Network (DCE-Net), which is responsible for estimating the enhancement curves, which are then applied to each color channel. The models can enhance low-light images but fail at transferring these results onto LLVE and some real-world low-light images. Both models fail at retaining semantic information and may often lead to unintended results such as over enhancement.

**Figure 7:**
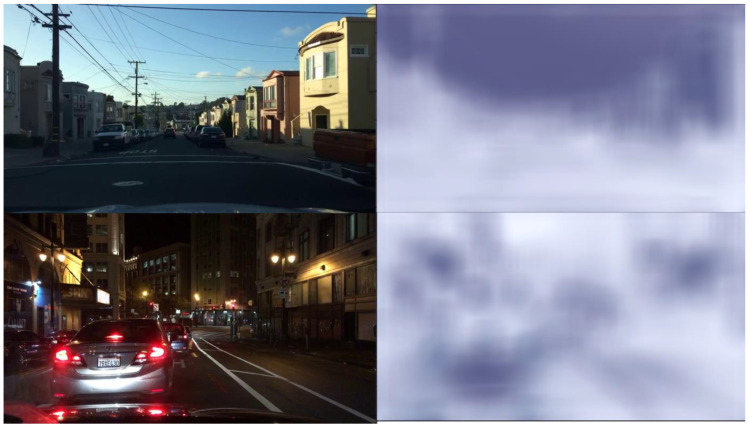
EFEN. Input images (**left**), pixel-wise light deficiency (**right**). (Reprinted from [39]).

To tackle the issue of semantics, Semantic-Guided Zero-Shot Learning (SGZ) for low-light image/video enhancement was proposed by [39]. The model proposes an enhancement factor extraction network (EFEN) to estimate the light deficiency at the pixel level, illustrated in Figure 7. The model also proposes a recurrent image enhancer for progressive enhancement of the image, and to preserve semantics, an unsupervised semantic segmentation network. The model introduced the Semantic Loss function, seen in (22), to maintain semantics, where HW are the height and width of the image, respectively, p is the segmentation network’s estimated class probability for a pixel, while β and γ are the focal coefficients. Although the introduction of the EFEN is critical and should be used in future research to guide other models for better pixel-wise light enhancement, the model still suffers from some challenges. The model performed poorly on video enhancements, resulting in flickering in the videos due to its overreliance on image-based datasets and lack of a network that takes advantage of frame neighbor relations.

(22) $$L_{sem\;}=\frac{1}{HW}\sum_{1\leq i\leq H,1\leq j\leq W}\;-\beta {\left(1-p_{i,j}\right)}^{\gamma }logp_{i,j}$$

Table 4 [40] compares various zero-shot enhancers to each other on various popular testing datasets. The models are compared on the Naturalness Image Quality Evaluator (NIQE), which is a non-reference image quality assessment, first formulated by [41].

### 2.4. Traditional Learning-Based Enhancers, a Deepe Dive

Traditional techniques were dominant pre-deep learning models and relied on traditional digital image processing techniques and mathematical approaches. This involved techniques such as histogram equalization, gamma correction and retinex theory.

Histogram equalization aims to improve the image quality by redistributing the pixel intensity to achieve a more uniformly distributed pixel intensity [42]. The method works well with global enhancement or suppression of light but does destroy the contrast relationship between local pixels. Given a greyscale image *I* = *i*(*x*,*y*) with *L* discrete intensity levels, where *i*(*x*,*y*) is the intensity of the pixel at coordinates (x,y) and *L* ∈ [0, *L* − 1], to histogram-equalize *I*, the probability distribution function is first obtained, which maps the distribution of each pixel intensity for the image. The cumulative distribution function (cdf) is next obtained, after which a transformation function is defined using the original cumulative distribution function as mathematically illustrated in (23).

(23) $$f\left(I\right)=I_{o}+\left(I_{L-1}-I_{o}\right)\times cdf(I_{x})$$

**Figure 8:**
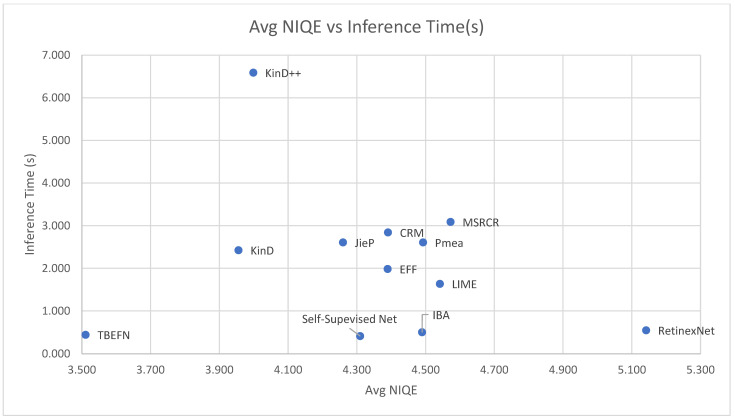
Average NIQE vs. inference time of models discussed in Table 5.

Retinex theory [3] separates an image into two components, a reflection map and illumination map. The reflectance component remains the same regardless of lighting conditions and is thus considered an intrinsic property, while the illumination map is a factor of the light intensity in the original image. The objective, therefore, is to enhance the image by enhancing the illumination map and fusing it with the reflectance map. The image and the two components of the image are illustrated formulaically in (24), where · is the element-wise multiplier. It should be noted that many deep learning methods [31,32,33,34,35,43,44] still borrow from the ideas of traditional learning techniques like Retinex theory. A quantitative comparison is provided by [45], Table 5, on such deep learning models and those (deep learning models) that do not adopt techniques from Retinex theory. Figure 8 compares the average NIQE score and inference times of some of the enhancement networks explored in Table 5. In Figure 8, TBEFN is observed to have a low inference time and low NIQE score, which are the desired conditions.

(24) $$Image\left(x,y\right)=Reflectance\left(x,y\right)\cdot Illumination(x,y)$$

### 2.5. Unsupervised Learning-Based Enhancers

Unsupervised learning LE models do not require paired data, but rather low-light and optimally lit images of different scenes can be “paired”. Such models have the edge over supervised models as less time is wasted mining the data (since the data are easier to acquire) while also benefiting from having pseudo-paired data which allow supervised models to outperform zero-shot learners.

**Figure 9:**
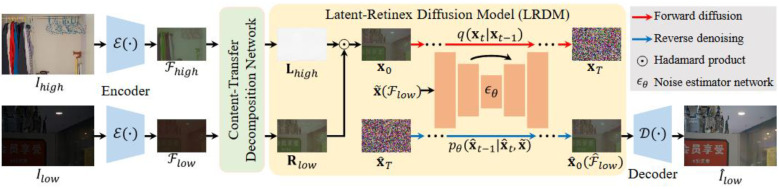
LightenDiffusion pipeline. (Reprinted from [56].)The Hadamard product is the element-wise product. The Encoders transform input images (both low-light and normal lighting) to their latent representations (F). These representations are then sent to the Content-Transfer Decomposition Network to generate content-rich reflectance maps (R) and content-free illumination maps (L). The maps matrices are multiplied elementwise after which the Hadamard product is sent to the forward diffusion network and reverse denoising network.

LightenDiffusion [56] proposed an unsupervised light enhancer which is based on diffusion while also incorporating Retinex theory. To improve the visual quality of the enhanced image, ref. [56] performs Retinex decomposition on the latent space instead of the image space. This allows for capturing high-level features such as structural, context and content features. The raw pixels are also sensitive to noise and thus the amplification of this noise is avoided, which, as previously stated, is a problem most enhancers still need to solve. Figure 9 pictorially illustrates the model. The unpaired low-light and normal-light images are first fed into an encoder, which converts the data into their latent space equivalence. The outputs of the encoder are then fed to a content-transfer decomposition network, which is responsible for decomposing each of the latent representations into their illumination and reflectance mappings. Next, the reflectance map of the poorly lit image and illumination of the optimally lit image are fed into the diffusion model which performs the forward diffusion process. Reverse denoising is performed to produce the restored feature flow (${\hat{f}}_{low}$) which is sent to the decoder, which restores the low-light image to the target image.

Table 6 presents a quantitative comparison between unsupervised models. The results are obtained from results obtained from [56]. The models are evaluated on four metrics previously introduced and the Perception Index (PI) [57], which has yet to be discussed in this paper. The PI combines two non-reference metrics, the NIQE and Perceptual Quality Score [58].

**Figure 10:**
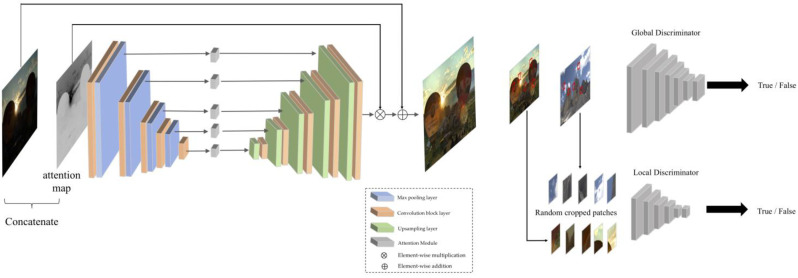
Architecture of EnlightenGAN. (Reprinted from [59].)

Another popular unsupervised model is EnlightenGAN [59], a generative adversarial network. EnlightenGAN is based on a GAN architecture with the generator being used to enhance images, while the discriminator aims to distinguish between the target and the enhanced images. EnlightenGAN also adopts Global-Local discriminators for enhancing not only the global features but also local areas such as a small bright spot in a scene, pictorially illustrated in Figure 10. Although the model is advantageous in that it does not require paired data and is able to adapt to varying light conditions while also enhancing both local and global features, a major hinderance of GANs, as noted by [60], is their instability and how they require careful tuning. GANs are particularly useful as they focus on perceptual quality and thus generally focus on producing results that are optimized for human perception.

**Table 6:** Quantitative comparison between unsupervised models as reported by [56], where higher values for PSNR and SSIM are desired while lower values for NIQE and PI are desired. Performance measure completed using LOL [35], LSRW [61], DICM [62], NPE [31] and VV [63] datasets. The best scores are in bold. (Reprinted from [56].)

Model	LOL		LSRW		DICM		NPE		VV	
	PSNR	SSIM	PSNR	SSIM	NIQE	PI	NIQE	PI	NIQE	PI
EnlightenGAN [59]	17.606	0.653	17.11	0.463	3.832	3.256	3.775	2.953	3.689	2.749
RUAS [64]	16.405	0.503	14.27	0.461	7.306	5.7	7.198	5.651	4.987	4.329
SCI [65]	14.784	0.525	15.24	0.419	4.519	3.7	4.124	3.534	5.312	3.648
GDP [66]	15.896	0.542	12.89	0.362	4.358	3.552	4.032	3.097	4.683	3.431
PairLIE [67]	19.514	0.731	17.6	0.501	4.282	3.469	4.661	3.543	3.373	2.734
NeRCo [68]	19.738	0.74	17.84	0.535	4.107	3.345	3.902	3.037	3.765	3.094
LightenDiffusion [56]	**20.453**	**0.803**	**18.56**	**0.539**	**3.724**	**3.144**	**3.618**	**2.879**	**2.941**	**2.558**

### 2.6. Semi-Supervised Learning-Based Enhancers

Semi-supervised enhancers make use of both paired and unpaired data, although the data distribution is often skewed. Semi-supervised enhancers contain small amounts of paired data while utilizing large amounts of unpaired data.

**Figure 11:**
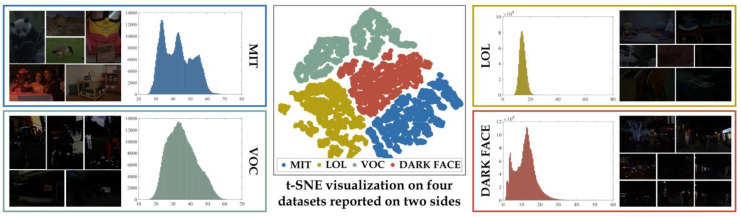
t-SNE [71] of common training datasets in the LLE domain, which highlights the distribution discrepancy of said models. (Reprinted from [69]).

Ref. [69] proposed an enhancer which aims to tackle the issue of previous enhancers being unable to generalize to the real world, thus resulting in modeling performing well on scenes similar to those found during training but struggling when new scenes, with never before seen lighting conditions and noise patterns. Ref. [69] identified that the training data used for enhancers were not diverse enough in lighting conditions and noise patterns; rather, previous enhancers used different scenes, but the lighting conditions from one pair to another were not diverse enough. This lack of diversity is shown in Figure 11 [69], where popular training datasets’ t-SNE distributions are visualized. This visualization shows how the popular practice of only training a model on one of these datasets limits its ability to generalize to other data and potentially the real world.

Bilevel Fast Scene Adaptation [69], learns to generalize representations of various diverse datasets and uses this knowledge to train an encoder to be scene-independent. To further adapt to new scenes and reduce computational requirements, when new data are encountered, the encoder is frozen and only the decoder is fine-tuned.

In the domain of LE, semi-supervised learning is the least utilized strategy, and thus a limited pool of models exists to compare one to another. Table 7 compares Bilevel Fast Scene Adaptation to a popular semi-supervised LE model, the Deep Recursive Band Network (DRBN) [70].

### 2.7. Mixed Approach

Ref. [72] proposed the use of a mixed algorithm, DiffLight, which leverages the advantages of the already discussed techniques. DiffLight utilizes the local feature extraction capabilities of CNNs, global dependency modeling of transformers and abilities of diffusion models to generate new data. DiffLight also combines multiple loss functions to balance perceptual quality and accuracy. LPIPS is used to provide image quality from the perspective of a human being (quality based on human perception). LPIPS is the Learned Perceptual Image Patch Similarity and compares the perceptual similarity between two images. First proposed in [73], LPIPS is the measurement of the distance between the two images (25), and thus lower values imply that the produced image is close to the desired image. Table 8, as reported by [72], compares [72] with some of the models it adopts from, and a comparison is performed using LPIPS, SSIM and PSNR on the LOLv1 dataset.

(25) $$d_{LPIPS}(x,y)=\sum_{l}\;w_{l}\cdot {\left\|\phi_{l}(x)-\phi_{l}(y)\right\|}_{2}$$

where

- x is the reference image, and y is the distorted image.
- $\phi_{l}$ is the feature map extracted from l-th layer
- $w_{l}$ is the weight of the l-th layer
- ‖◦‖ denotes the Euclidean distance.
· denotes the elementwise multiplication

Another algorithm that adopts a mixed approach to low-light image enhancement is [81], which inherits the strengths of wavelet transformations and diffusion-based generative modeling. The wavelet-based diffusion framework enhances the image illumination and preserves detail while the diffusion process enhances image quality through noise filtration. The model makes use of wavelets to ensure efficient use of resources as compared to other vanilla diffusion models.

### 2.8. Loss Functions

#### 2.8.1. Pixel-Level Loss Functions

The Mean Squared Error (MSE) Loss and Mean Absolute Error (MAE) Loss both operate at the pixel level. These errors are simple to implement and compare the pixel values of the predicted output to the target output, and as a result, are commonly implemented in supervised enhancers. The MSE is able to penalize large errors, but is sensitive to outliers, and as noted in [82], in light enhancement, this may result in noise amplification. The MAE is more robust to outliers than the MSE but, in light enhancement, may lead to loss of detail and perceptual quality.

#### 2.8.2. Perceptual Loss Functions

The Perceptual Loss maintains high-level details that the pixel-level losses are unable to maintain. Perceptual Loss focuses more on ensuring that the image appears natural to the human eye and thus is widely used in the light enhancement domain.

The Naturalness Image Quality Evaluator (NIQE) is another Perceptual Loss function, with its major differentiating factor being that it is a no-reference loss function, making it a major player when it comes to unsupervised enhancers. NIQE makes use of statistical regularities that are present in natural images and therefore does not require a reference image.

#### 2.8.3. Color Loss Function

The Color Constancy Loss maintains RGB channel ratios to ensure that color constancy is maintained regardless of changing illumination, making the loss function very popular with light enhancement algorithms.

#### 2.8.4. Smoothness Function

Due to the presence of noise in low-light data, and how during enhancement, light enhancement algorithms often enhance this noise further, the Total Variation (TV) loss has become a popular stable of said algorithms. The TV loss enforces spatial smoothness in neighboring pixels, thus reducing noise in the output image.

## 3. Artefact Removal Networks

### 3.1. Deblurring Algorithms

The blurring of an image/video frame can be formulated by (26) [83], where $I_{b}$ is the blurred frame, Φ is the blurring function, $\theta_{\eta }$ is a parameter vector and $I_{s}$ is the desired sharpened frame [83]. Therefore, for deblurring, the goal is to find the inverse of (26).

(26) $$I_{b}=\Phi \left(I_{s};\theta_{\eta }\right)$$

#### 3.1.1. Non-Blind Deblurring

**Figure 12:**
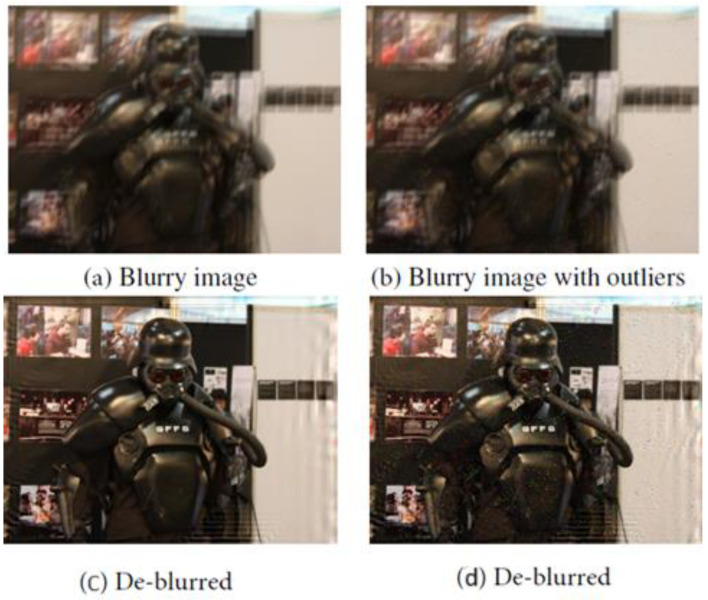
(**a**–**d**) Ringing artefact in noisy deblurred image. (Reprinted with permission from [84], 2022, IEEE., 3 Park Avenue, 17th Floor, New York, NY 10016).

Since many non-blind deblurring methods are sensitive to filter noise, ref. [84] introduces two error terms to prevent ringing artefacts in the deblurred image. The first error term is called “Kernel Error” and approximates the error of the kernel produced by the deblurring methods. The second error is termed “Residual Error” and is introduced to handle noise in blurry images, without which (residual error) the deblurred image contains ringing artefacts around the noisy locations, observed in Figure 12. Finally, to recover the desired image data, a denoiser prior is adopted. The algorithm is modeled by (27), where k is the returned kernel by the deblurring method, and t is the kernel error term, while s is the residual error term. β and λ are adjustable parameter terms and the implicit denoiser prior is denoted by Φ(·).

(27) $$\underset{x,t,s}{min}\;\frac{1}{2}∥\left(k+t\right)\times x-y-s{∥}_{2}^{2}+\lambda \Phi (x)+\frac{\alpha }{2}∥t{∥}_{2}^{2}+\beta ∥s{∥}_{1}$$

**Figure 13:**
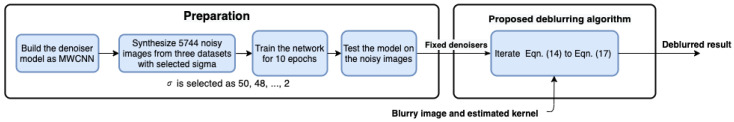
Model’s training and testing flowchart. (Reprinted from [84]).

Figure 13 represents the model’s flowchart for training and testing procedures, with “iterate Equations (14)–(17)” being the process of solving, iteratively, the formulated model in (27). A major drawback of the model is that the Multi-level Wavelet-CNN (MWCNN) model is trained with a specific level of Gaussian Noise, and thus for varying noise training, a series of MWCNNs is needed. The model’s inability to generalize to noise makes it unsuitable for real-word applications.

#### 3.1.2. Blind Deblurring

DeblurGAN [85] leverages Conditional Generative Adversarial Networks (cGANs) to recover sharp images from motion blurred ones. The model uses a CNN generator and a Wasserstein GAN with a gradient penalty. The model formulates a loss function for achieving state-of-the-art sharp images. The loss function used is a summation of the adversarial loss and content loss, where the Perceptual Loss is adopted as the content loss instead of the classical L1 or L2 losses. The model highlighted the benefits of using both synthetic and real data as opposed to just one or the other as improved model results were observed when a combination was used. The model is, however, hindered by its non-real-time inference speed of 0.85 s. Furthermore, in a benchmark study [86], DeblurGAN showed poor results as compared to other models when it comes to real-world tests. Its successor, DeblurGAN-V2 [87], showed great improvement and was amongst the best models in the same study [86], but ref. [88] showed that DeblurGAN-V2 achieved much slower inference times; thus, the DeblurGAN family is plagued by non-real-time inference times and poor real-world data performances.

Xiang et al. [89] performed a deep and comprehensive survey on blind deblurring methods, focusing on four deep learning networks, based on CNNs, RNNs, GANs and transformers. The researchers found that transformer-based methods were the best-performing methods, with the FFTformer and Uformer being the best-performing models tested on four image deblurring datasets, GoPro [90], HIDE [91], RealBlur-J [86] and RealBlur-R [86]. However, transformers are still a new concept in the world of computer science and more work is needed to make them less computer resource-intensive and more efficient. CNN- and RNN-based algorithms are the next best methods according to [89], but each method does come with its drawbacks. CNN-based methods are unable to handle long-range dependencies, such as with videos and dynamic frames, and as such, RNNs are utilized for such cases. RNNs are known to suffer from exploding and vanishing gradients when training, which leads to the poor performance of the models during testing. The gradient problems of RNNs can be solved in various ways, either by utilizing Gated Recurrent Units (GRUs), gradient clipping or ResNets.

#### 3.1.3. Non-Uniform Deblurring (Local Deblur)

**Figure 14:**
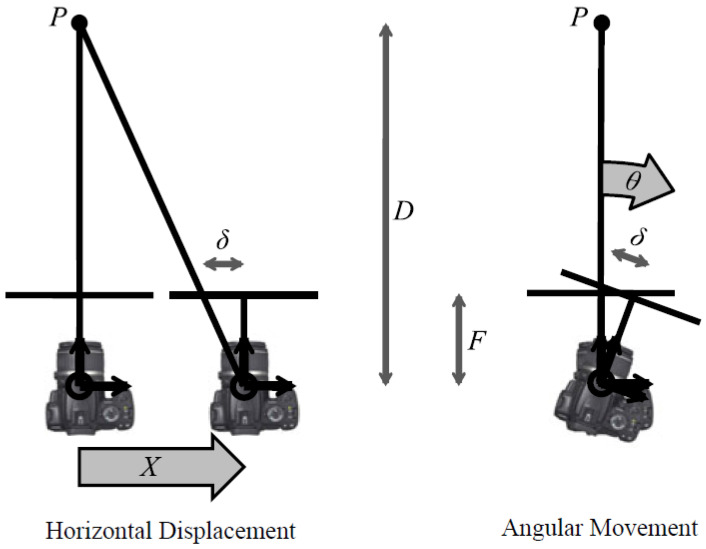
Camera shake. (Reprinted from [93]).

To capture non-uniform blur in an image due to camera shake, ref. [92] proposed a parametrized geometric model. The proposed network aims to show that camera shake is, in most cases, a result of the camera’s 3D rotation. The model’s advantages are that it can be easily integrated into any existing deblurring networks with minor modification and that it employs a single global descriptor. The 3D movement of the camera is modeled by (28), which represents the camera’s horizontal displacement, and (29), which represent the camera’s angular movement; these are pictorially illustrated in Figure 14.

The algorithm is hindered by its bloated runtime, where a single image can take several hours to deblur, thus making it impractical for real-time applications. The model is also limited in its ability to deblur angular movement, being able to only deblur rotations of 3° to 5° inclusive.

(28) $$X=\frac{\delta }{F}D$$

(29) $$\theta =\tan^{-1}\left(\frac{\delta }{F}\right)$$

#### 3.1.4. Uniform Deblurring

Uniform deblurring is often applied in instances where the cause of the blur is a single constant source, usually the camera. Since cameras capture images by measuring photon intensities over a period of time, in order to capture a sharp image, this period of time needs to be short, as longer time periods, also known as long exposure, will lead to a blurry image [83]. Since blurs are nothing more than convolutions of a kernel (known or unknown) on a sharp image, deconvolution methods are utilized to obtain the sharp image, such as FFT-based methods. Kruse et al. [94] introduced generalized efficient deconvolution methods based on FFT, employing CNN-based regularization. The model generalizes shrinkage fields through the elimination of redundant parameter sharing and replaces pixel-wise functions with CNNs that operate globally. The corrupted image (y) can be modeled as a result of the convolution of the kernel (k) and sharp image (x) illustrated in (30). Kruse at al. [94], using CNNs, modeled (30) replacing shrinkage functions, where $F^{-1}$ is the IFFT, $\phi_{t}^{\mathrm{CNN}}$ is the CNN-based learned term, $f_{\mathrm{it}}$ are the linear filters and the learned scalar function that acts as a noise-specific regularization weight is denoted by $\omega_{t}\left(\lambda \right)$. The algorithm proposed in [94] suffers from non-real-time inference speeds. The model’s inference time is heavily dependent on the size of the image it is processing, in some instances producing results in 0.75 s.

(30) $$x^{t+1}=F^{-1}\left(\frac{F\left(k⊛y+\frac{1}{\omega_{t}(\lambda )}\phi_{t}^{CNN}\left(x^{t}\right)\right)}{|F(k){|}^{2}+\frac{1}{\omega_{t}(\lambda )}\sum_{i}\;{\left|F\left(f_{it}\right)\right|}^{2}}\right)$$

Table 9a–c highlight the models discussed in this chapter. For comparison of each model’s performance, the same blurring kernel needs to be applied to all the models to ensure fair evaluation, and since not all models operate on the same kind of blur (i.e., uniform vs. non-uniform), algorithms that operate on the same blur need to be compared with each other. Refs. [88,95] provide some comparisons between more algorithms.

Table 9c presents the self-reported results of the artefact removal models. Ref. [84] presents the average results from Levin’s dataset [98]. Two models were investigated in [94], a greedily trained model, and one fine-tuned model, which is initialized by the greedy model. The model presented in Table 9c is the fine-tuned model, tested on Levin’s dataset [98]. The results from [85] were tested on the GoPro [90] dataset. Ref. [87] used the Inception-ResNet-v2 backbone and tested it on GoPro [90]. Ref. [96] was tested on the GoPro. Ref. [97] was tested on the GoPro dataset.

### 3.2. Color Grading and Flicker Removal Algorithms

Poor colorization and flickering in videos are related, where video flickers can be observed as brightness changes in neighboring video frames, and thus can be tackled with the same algorithms. Traditionally, the issue of flickering can be simply tackled by using a non-degraded neighboring frame in the video to reconstruct other frames [99]. This solution fails for general purpose cases as noted in [99], such as when two neighboring frames experience scene change.

**Figure 15:**
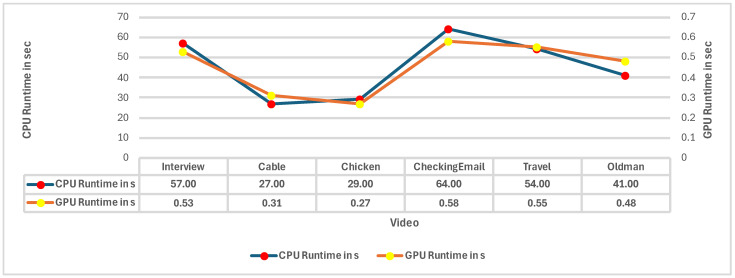
Runtimes on CPU and GPU. (Reprinted from [99]).

Hence, ref. [99] proposed a multi-frame-based method for video flicker removal, where the frames used for reconstruction are first evaluated for temporal coherence. The benefit of this method is the use of multiple spatiotemporally coherent frames for reconstruction, ensuring that scene changes are accounted for in the reconstruction algorithm. The model uses simple linear iterative clustering (SLIC) to divide an image frame into superpixels (large pixel clusters of similar brightness and color), after which dense correspondence between frames is obtained using SIFT Flow (analogous to optical flow). The model is limited by numerous factors, one of them being its runtime, making it a non-real-time model. Figure 15 compares the performance of the model when tested on a CPU as well as a GPU. The results show that the model tested on a GPU has a runtime two orders of magnitude less than the one on a CPU, implying that for deep learning algorithms, a focus on hardware to increase inference times is just as vital as the focus on the algorithms themselves. Although the model attempts to use multiple spatiotemporally coherent frames to tackle issues related to objects being present or absent in neighboring frames, the model still fails at capturing spatiotemporal coherence on extremely dynamic frames and is limited to handling videos with few slow-moving objects. Finally, as part of the input data, the model requires the original non-flickering video, resulting in the model not being applicable for real-world blind deflickering.

**Figure 16:**
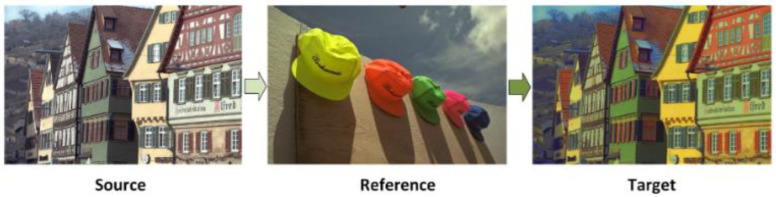
Example of good color transfer. (Reprinted from [100]).

**Figure 17:**
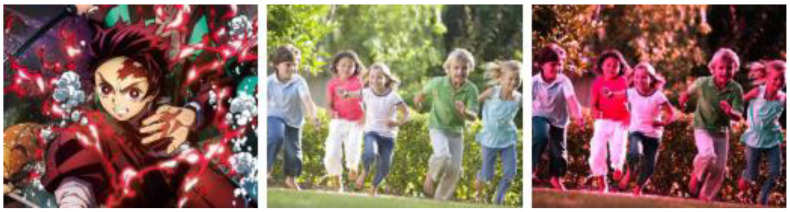
Poor color transfer. (Reprinted from [100]).

Color grading can be used for LLE, but color grading exhibits limiting factors which make it more suited for post LLE applications rather than being used for LLE directly. A major limiting factor being that color transfer requires a reference stage, where the input image is enhanced using a refence image with the same color schematic as the target image, as exemplified in Figure 16. If the color schemes of the source and reference images do not match, the target will not be obtained, Figure 17.

To tackle the issue of a specific reference image being required as input which the user may not know, ref. [100] introduced concept transfer, which can be used as input alongside the image to be graded. Lv et al. [100] defined concepts as terms like “romantic”, “earthy” and “luscious”. The model is useful in cases where the user does not know the correct reference image to use but is aware of the “concept” they wish to transfer to the enhanced image. The model is not too dissimilar to modern day image filters that operate by the user picking a name which is associated with a certain color pallet rather than transferring color by individually picking colors to transfer to the image.

### 3.3. Deep Video Prior

**Figure 18:**
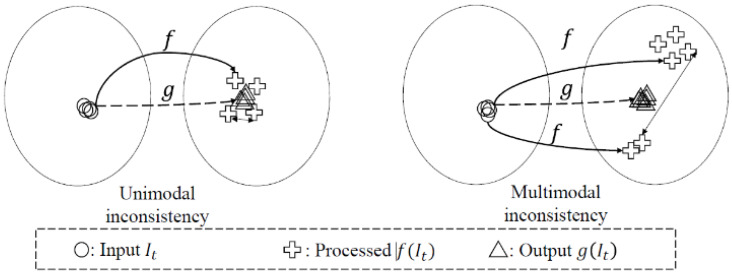
Illustration of unimodal and multimodal inconsistency. (Reprinted from [101].)

Lei [101] proposed deep video prior (DVP) to enforce temporal consistency in inconsistent videos, due to frame-by-frame processing. Several video processing techniques process videos frame-by-frame, thus treating them as a series of disconnected images, which ignores their temporal co-dependency. DVP is built upon Deep Image Prior (DIP) [102], which performs image denoising, image inpainting and super-resolution. DIP takes in as input random noise and is trained to reconstruct a sharp image. The work of DVP hypothesizes that flickering in the temporal domain is equivalent to noise. DVP also has the benefit of requiring only a single training video. An inconsistent video is formed when a temporally consistent input is mapped to an inconsistent output, causing artefacts. This inconsistency can be unimodal or multimodal, where multiple solutions exist for an object in the processed frames (e.g., an object that should be blue changes to orange and yellow). As illustrated in Figure 18, f is a random function that inconsistently maps the input to the output, while g is the random function that consistently maps the input to the output. DVP [101] proposes a fully convolutional network, g, that mimics the operation of the inconsistent function, f, while maintaining consistency. During training, the model uses a loss function which measures the distance between the mapping of g and f, and stops training when the distance approaches a minimum at which g would overfit to f, producing the same artefacts observed in f. The CNN (g function) adopts a U-Net and a Perceptual Loss for the loss function but is not limited to these adopted strategies. DVP is hindered by its non-real inference time, where single frames can average an inference time of 2 s.

## 4. Artefact Removal Coupled with Light Enhancement

As highlighted by [103], “Images captured under low-light conditions usually co-exist with low light and blur degradation”. This occurrence is a result of how a camera captures data. Under poor lighting conditions, the exposure time of a camera is longer in order to allow for more light into the camera for clearer images. This longer exposure time means that blurs are more likely to occur. This implies that the artefact removal techniques discussed in the previous chapter are limited to images and videos captured in daytime lighting conditions. Thus, to design a computer vision network that is robust enough to be functional in a variety of lighting conditions, joint low-light enhancement with deblurring is required. To emphasize this statement, the coupled and uncoupled artefact removal techniques will be compared to each other to evaluate how they perform in various conditions.

**Figure 19:**
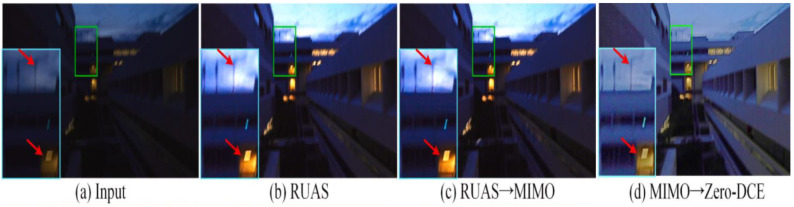
Effects of light enhancement cascaded with deblurring. The differences between the input blurry images and the output images of each model (**b**–**d**) can be seen in the blue and green squares. In (**d**) the poles as indicated by the red arrow are clearer as compared to (**b**,**c**). (Reprinted with permission from [103], 2024, Elsevier., Radarweg 29, 1043 NX Amsterdam, The Netherlands).

Ref. [103] proposed “joint low-light enhancement and deblurring with structural prior guidance”. The paper noted that there are two common approaches adopted when pursuing low-light enhancement and deblurring, cascaded light enhancement flowed by deblurring, and cascaded deblurring followed by enhancement. The first approach of enhancing the image before deblurring often causes saturated areas in the image to be overexposed; once overexposed, details of these areas become hidden and therefore the deblurring network is unable to deblur these areas, in some cases making them worse, as seen in Figure 19, where the input is a low-light blurred image, and the output (b) is the enhanced image, where enhancement was performed using RUAS [64]. The performance of the cascaded model (c), where RUAS [39] was cascaded with MIMO [104], is comparable with the input, with some areas incurring more blurs.

The alternative approach (deblurring followed by enhancement) often involves employing deblurring algorithms that are not trained on low-light data and are therefore poorly interpreted motion information and features in low-light conditions. Figure 19d shows the deblurring algorithm [40] cascaded with [8]. In both Figure 19c,d, the only major improvement from the input is the light enhancement, implying that designing a robust deblurring algorithm for nighttime operations is a non-trivial solution.

The proposed solution by [103] employs structural priors, a transformer backbone for capturing global features of the image. To compensate for the transformer’s poor capabilities at capturing local features, the images are also processed at smaller variable-sized blocks. This assists the model in understanding the ambiguity of blurs, and more specifically non-uniform blurs. Through the use of statistical methods, priors and parallel attention, the model attempts to recover the sharp (deblurred) and enhanced images. This proposed network is akin to the U-net, incorporating an encoder–decoder architecture and a feature reconstruction subnet. Shallow image features are extracted by the encoder, while feature refinement is handled by the decoder. Due to research conducted by [105], which showed that the Pyramid Pooling Module can depress noise in low-light enhancement, multiple layers are equipped with PPMs in the proposed model. Depth image features are captured in the feature reconstruction subnet, which are then sent to the SNR transformer [106]. The non-uniform blurs are handled in the multi-patch perception pyramid block while a proposed prior guided reconstruction block adaptively reconstructs the enhanced sharp image.

The model algorithm is summarized in Algorithm 1.
**Algorithm 1** Joint low-light enhancement and deblurring algorithm. (Reprinted from [103]).**Input**: low-light blurry image *I_in_***Output**: normal-light sharp image *I_out_*1: Extract shallow image features ℱ*_e_* with encoder2: Enhance image features ℱ*_e_* using the feature reconstruction subnet, where the SNR Transformer strengthens global features ℱ*_i_* and the MPP block generates structural features ℱ*_i_^h^*, ℱ*_i_^e^*3: Fuse image features ℱ*_i_* and structural features ℱ*_i_^h^*, ℱ*_i_^e^* through the PR block4: Decoder refines and restores the normal-light sharp image *I_out_*5: **return** *I_out_*


Table 10 compiled from [103] is a quantitative comparison of the proposed model in [103], and cascaded deblurring and light enhancement models as well as uncascaded light enhancement and deblurring that were retrained on the LOL-Blur dataset [76]. The results obtained by the authors confirm that simply cascading light enhancement and deblurring for better performance is not advisable. The uncascaded and retrained light enhancement and deblurring network performs better than the cascaded models but the best-performing models are algorithms that perform joint light enhacmenet and deblurring. The best-perfoming model is highligted in red, while the next best is blue. Although the model in [103] is able to enhance light, it is unable to fully recover the sharp images. The model’s bloated nature results in the model having 40.1 M parameters and having a runtime of 10 s [103], restricting it to offline use.

**Figure 20:**
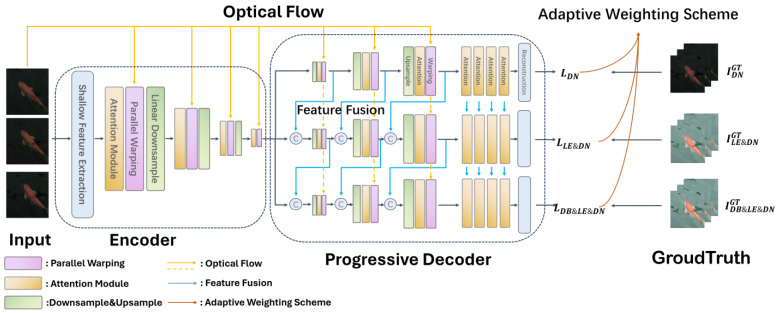
VJT illustration with encoder and multi-tier decoder. (Reprinted from [109]).

Video Joint Task (VJT) [109] aims to tackle the combined challenge of deblurring, enhancing low-light images and denoising by proposing a three-tiered decoder structure which steadily fits to different levels of the desired results. For facilitating the transition from shallow to deep learning, a feature fusion module is designed. The input to the model, shown in Figure 20, is the undesired video sequences which are fed into the encoder. In the encoder, shallow features are extracted along with frame alignment through warping and an attention module which focuses on the key parts of the video (noisy areas, moving objects). The video data’s spatial dimensions are reduced through downsampling and this reduced video data are the input to the progressive decoder. The feature fusion (in the decoder) ensures that features from different layers are present in the final output. Different weights are assigned for the various outputs depending on the task (restoration, enhancement, etc.) by the adaptive weighting scheme. The adaptive weighting scheme builds on research by [110]. The adaptive weighting scheme addresses the challenges of multi-task loss, ensuring that the contribution of each loss is dynamically balanced. The three losses that are dynamically balanced are the denoising loss, the combined low-light enhancement and denoising loss and a loss for integrated deblurring, enhancement and artefact removal. The model is trained on the dataset created by the authors, the Multi-scenes Lowlight-Blur-Noise Dataset (MLBN) [109], which contains indoor, nighttime outdoor and daytime outdoor scenes. The dataset is a mixture of real and synthetic data, where motion blurs, low illumination and noise are synthetically added.

The researchers performed a quantitative analysis on the proposed methods against other deblurring methods. The results, Table 11 [109], show that just as seen in image processing, in video processing, a joint approach (light enhancement and deblurring) is beneficial for robust denoising systems. VJT (joint approach) is compared with purely deblurring models. The best-performing model is indicated in bold.

## 5. Action Recognition and Object Detection

### 5.1. Action Recognition

**Figure 21:**
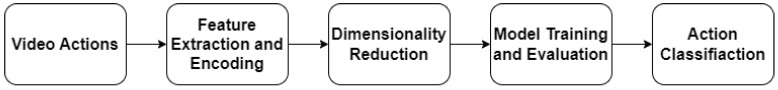
AR pipeline.

Action recognition (AR) is explained simply by [115] as an attempt “to understand human behavior and assign a label to each action”, and Figure 21 maps out the standard pipeline for AR.

**Figure 22:**
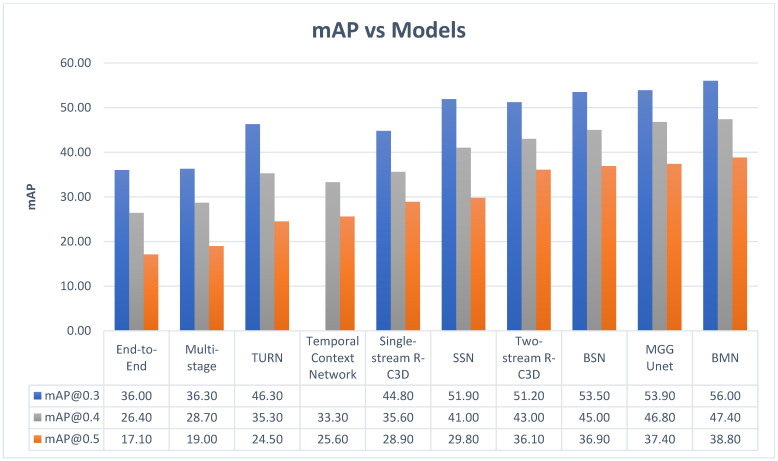
Mean average precision of various AR models, obtained from [117], using the THUMOS’14 [118] dataset.

Pareek and Thakkar [116] conducted a comprehensive review of current and future trends in action recognition. According to [116], the major challenges facing traditional methods are feature extraction and training time. As most traditional methods of AR employ supervised learning, the feature extraction involves manually labeling the data. In the case of video actions, this will require large amounts of time, and as the dataset grows, so does the time spent extracting features. The solution to this problem may lie in zero-shot learning, which, unlike other learning methods, can classify new classes without prior knowledge of those classes, relying purely on semantics. Although deep learning (DL) methods remove the need for manual feature extraction and generally perform better than traditional methods, ref. [116] noted that some DL methods, like RNNs which are useful for processing sequential data like videos, can suffer from exploding or vanishing gradients. LSTMs, which are part of the RNN family, do not suffer from the gradient issue, but are unable to capture spatial information which is important for action, actor and object associations. Table 12 summarizes other models in the action recognition domain, while Figure 22 numerically compares the performance of the models discussed in Table 12. In Figure 22, the mean average precisions at different thresholds are obtained from [117], using the THUMOS’14 [118] dataset.

### 5.2. Object Detection

“Object detection involves detecting instances of objects from one or several classes in an image” [119]. Object detection algorithms are divided into either one-stage detectors or two-stage detectors, where one-stage detectors are faster while two-stage detectors are more accurate. As one-stage detectors prioritize speed over accuracy, they are usually implemented for real-time applications while two-stage detectors are usually used for offline medical or crime analysis where time is not as important as accuracy. The difference between the two algorithms is that the single stage moves directly from extracting features from the input image to outputting bounding boxes and classifications. In the case of a two-stage detector, an additional stage of region proposals is added, which outputs proposals of the regions which are most likely to have objects; the model then slides over these proposed regions to classify the objects instead of the entire image.

A. One-Stage Detectors

**Figure 23:**
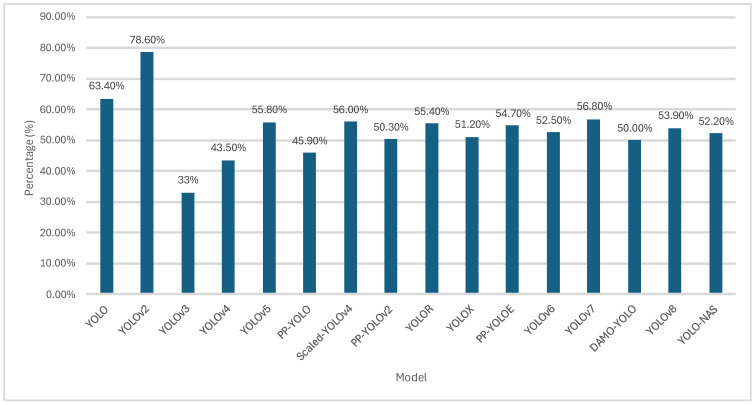
Average precision of various YOLO models. (Reprinted from [120].)

Table 13 summarizes the various YOLO models over the years. The benchmark dataset for YOLO and YOLOv2 was the VOC2007 [22], while the rest were reported on COCO2017 [120]. The YOLO algorithm is limited as it can only make a single prediction per S×S cell, meaning that for smaller closely packed objects, the model struggles. The solution to this would be to divide the image into smaller boxes, which would then slow down the model; thus, there exists a trade-off between detecting small closely packed objects and speed. Figure 23 [120] shows the average precision achieved by various YOLO models using the datasets forementioned.

B. Two-Stage Detectors

**Figure 24:**
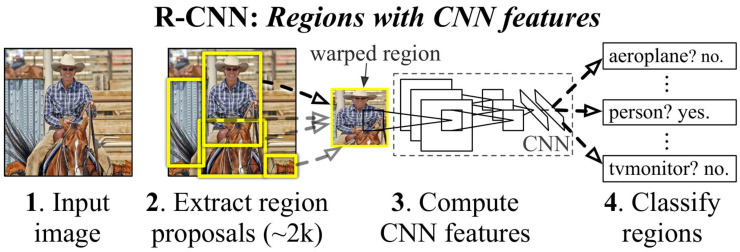
R-CNN structure. (Reprinted from [121].)

**Figure 25:**
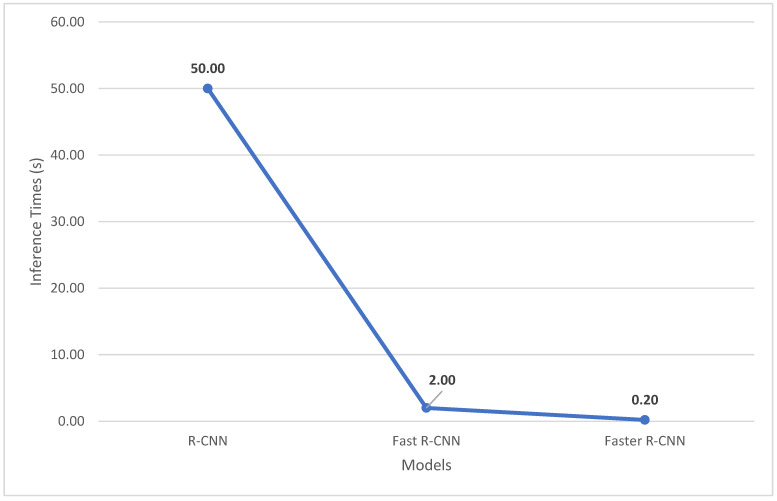
Inference times in seconds of various RCNNs. (Reprinted from [124]).

R-CNNs [121] remain amongst the most popular two-stage detectors, with the “R” standing for “Region”, for the region proposal that distinguishes two-stage from one-stage detectors. The algorithm contains three modules. The first region generates category-independent region proposals, which are the regions the rest of the model will use for object detection, ignoring the rest of the image. The next module is the feature-extracting CNN, which extracts fixed length features to feed to the final classification layer. The final layer contains a set of class-specific linear Support Vector Machines (SVMs), where each SVM is trained for the detection of specific objects and each SVM outputs its confidence for that specific object being present in each of the proposed regions, illustrated in Figure 24 [121]. Although the model has achieved state-of-the-art accuracy for detection of objects, its application is limited to offline as R-CNNs have an inference time of 40–50 s, illustrated in Figure 25.

Fast R-CNN [122] and Faster R-CNN [123] were proposed as improvements on RCNN. Fast R-CNN obtained its name for its fast training and testing time, along with the following advantages over R-CNN:

- Higher mAP as compared to R-CNN;
- Single-stage training and combines multiple objectives into a single “Multi-Task Loss”;
- Model can learn and refine features at every level.

## 6. Recognition and Detection Coupled with Light Enhancement

In order to evaluate the performance of their light enhancement model, ref. [8] tested the performance of a face detector on images enhanced by their model. The face detector algorithm chosen was the Dual Shot Face Detector (DSFD) and the dataset chosen was the DARKFACE dataset [125]. The dataset contains 10,000 low-light images. The purpose of the test is to illustrate how light has been enhanced to such an extent that a face detector can extract more useful information after enhancement. The test was performed alongside other low-light enhancers, but the detector remained unchanged. The input is the raw unenhanced data. As can be seen in Table 14 [8], the performance of all the enhanced images (regardless of the enhancement model) was better than the model tested on raw images, which emphasizes the need for an enhancer to allow detection models to function even under extreme conditions.

**Figure 26:**
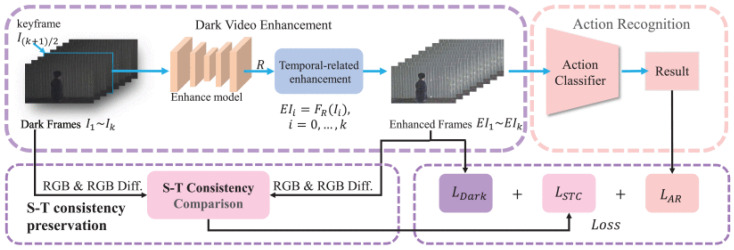
DTCM structure. (Reprinted with permission from [129], 2023, IEEE, 3 Park Avenue, 17th Floor, New York, NY 10016).

The Dark Temporal Consistency Model (DTCM) [129] (framework shown in Figure 26) proposes a joint approach for human action detection in dark videos with video enhancement. DTCM explores a cascaded approach where the video data are enhanced, after which human actions are deduced from the video. To ensure spatiotemporal consistency frame by frame, the model compares RGB differences before and after video enhancement. The model makes use of three losses.

The first loss (31) employed is the spatiotemporal consistency loss, which is a combination of the temporal and spatial consistency losses. The spatial consistency loss is formulated by (32), while the temporal consistency loss is shown as (33). The goal is to minimize (31), as the value of $L_{STC}$ represents the loss of inconsistency in the enhanced video. The temporal consistency loss, formulated in (33), is responsible for ensuring temporal constancy by comparing the RGB differences in the pre and post processing video. In (33), T denotes the duration of the input video clip, DY and DI are the enhanced frame and input frame, respectively. $\left|DY_{t+1}-DY_{t}\right|,\;\left|DI_{t+1}-DI_{t}\right|$ are the RGB differences of the enhanced and dark frames, respectively.

(31) $$L_{STC}=L_{SC}+L_{TC}$$

(32) $$L_{SC}=\frac{1}{T}\sum_{t=1}^{T}\;L_{SCF}\left(DY_{t},DI_{t}\right)$$

(33) $$L_{TC}=\frac{1}{T-1}\sum_{t=1}^{T-1}\;L_{SCF}\left(\left|DY_{t+1}-DY_{t}\right|,\left|DI_{t+1}-DI_{t}\right|\right)$$

The second loss employed by the model is the dark enhancement loss. The dark enhancement loss takes inspiration from Zero-DCE [8], and uses the loss functions defined in [8]. The dark enhancement loss (34) is a summation of the exposure control loss, the illumination smoothness loss and color constancy loss. The color constancy loss and the illumination losses have unique weights, while the exposure loss is unweighted.

(34) $$L_{\mathrm{Dark}\;}=L_{\exp\;}+W_{col}L_{col}+W_{tv_{A}}L_{tv_{A}}$$

For action recognition, the model makes use of the vanilla cross entropy loss

The model was tested on the UAVHuman-Fisheye Dataset, which contains distorted dark and light human action videos. As seen in Table 15 [129], the model is the best-performing action detection model amongst other state-of-the art detectors, which indicates that the model is not only suited for low-light action detection, but general action detection.

## 7. Datasets

### 7.1. LE Datasets

Zheng et al. [1] performed a comprehensive breakdown of the datasets available and their frequency of use in the LE domain. A summary of the most popular datasets is provided in Table 16. In Table 16, “size” represents the size of the dataset in terms of datapoints, while “paired” states whether each datapoint has its corresponding optimally lit pair. It was highlighted in [1] that numerous popular datasets in the LE domain fail to capture real-world conditions, where some objects in the image may be optimally lit, while others are over/under-illuminated. These lighting conditions can also exhibit gradual transitions in a single image rather than discrete luminosity level changes. For this, they proposed a new dataset with gradual transitions in luminosity within a single datapoint, along with another dataset with random transitions; the datasets were termed SICE_Grad [1] and SICE_Mix [1], respectively. Models trained on these datasets were observed to perform better for real-world conditions [1]. These observations reaffirm that for any DL algorithm, model design and configuration are just as important for better results as dataset quality.

### 7.2. Artefact Removal Datasets

In Table 17, “blur type” states what type of blur the dataset contains (uniform or non-uniform) and “domain” states what objects/items are present (scenes, human faces, objects), where scene describes a scene being blurred and no singular object being the focus of the datapoint. Most of the datasets in Table 17 synthetically generate realistic blurs using a method that has become popular recently, where blurs are created by averaging successive frames of videos.

### 7.3. Action Recognition and Object Detection Datasets

There exist a myriad of action recognition and object detection datasets. Table 18, on the other hand, summarizes the much harder to find criminal activity and weapons detection datasets. Many datasets pertaining to violent action recognition rely on synthetic data such as fights from movies or fights from sporting events. In Table 18, “Type” describes the type of crime (robbery, contact crimes, etc.) and “weapons” describes whether traditional weapons are used in any of the crimes (i.e., guns, knives are traditional weapons, whereas sticks, bats and rocks are not). The synthetic crime videos may contain either crimes acted out by researchers or criminal activities from movies scenes.

## 8. Challenges

Having surveyed research papers in the domain of LE, artefact removal, action recognition and object detection, three major problems have been identified that hinder progress in these fields:

- Lack of real-world datasets and data variability.
- Lack of benchmark models.
- Lack of real-time models.

### 8.1. Dataset Challenges

#### 8.1.1. Lack of Real-World Datasets

**Figure 27:**
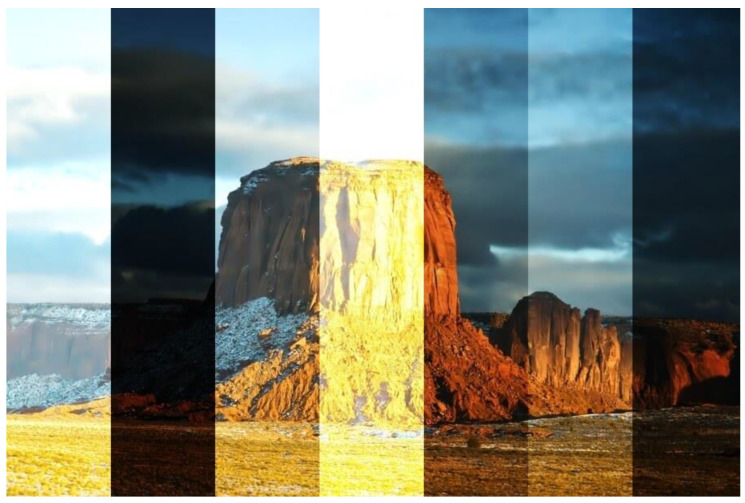
SICE_MIX example. (Reprinted from [1]).

There exists a severe lack of real-world datasets for the computer vision tasks described in this review. Since most LE models employ some form of guided learning, pairs of poorly lit and optimally lit images/videos of the same scene are required, but are hard to acquire. Researchers often resort to recreating these conditions indoors with and without artificial lighting such as with the popular Low-Light (LOL) dataset. This causes problems when the algorithm is required to be placed in real-world conditions, where lighting conditions are no longer binary, along with other lighting phenomena such as back-lighting, front-lighting and over-lighting. A proposed solution to this is the SICE_MIX [1] dataset, illustrated in Figure 27, which utilizes a mixture of lighting conditions in a single frame to imitate real-world lighting.

The same issue that plagues LE models is also present with deblurring algorithms. With deblurring algorithms, the method used to create artificial datasets is to apply a blurring filter over the entire image, usually a uniform one. The issue with this is that blurs are not often uniform in the real world; two objects moving at unique high speeds will, respectively, have unique blur kernels.

With violent action recognition, it is often difficult to find violent videos online due to various reasons such as victim protection, website policies against violent videos and poor quality of the data, as most violent acts occur at night in dark areas. This leads to some datasets containing mostly poor-quality videos, videos acted out by amateur actors, sport fights and even violent scenes from movies.

A solution to the issue of dataset availability is to use few-shot or even zero-shot learning, which has the advantage of being able to categorize classes that were not present during training while requiring little training data. Supervised learning is often preferred due to its more accurate results, but with the rise of unsupervised models like GPT-3 (which under zero-shot conditions have been shown to produce sufficient results), few-shot and zero-shot learners may be the dominant models of the future, thanks to how quick it is to build and train them, along with their adaptability to the real world.

#### 8.1.2. Biases in Datasets

A common issue that hinders deep learners is biases in the dataset used for training and testing. The first major bias is sampling bias, whereby the datapoints in the dataset do not reflect a true representation of real life. This phenomenon is observed in synthetic datasets such as the commonly used LOL dataset. This dataset contains mostly indoor paired images, where the low-light pairs are synthetically created. The synthetic pairs fail to capture the naturalness of real-world lighting conditions, which was discussed in Section 8.1.1. The models trained on these biased datasets thus perform well on the equally biased testing data but show a poor adaptability to real-world conditions. Overfitting bias is another problem that exists in low-light data collection. When designing a model, most of the data used are usually extremely dark, which overfits the model to such conditions. A good model is also trained with extremely bright images along with other conditions such as slightly dark images. Training a model with diverse data as described enforces the knowledge that some scenes may require little enhancement or no enhancement at all. This issue is illustrated in Section 2.6, where the t-SNE of commonly used datasets is shown, illustrating how choosing only one of these datasets for the design of a low-light enhancement model limits the model to learn limited characteristics about low-light data.

### 8.2. Benchmarking

Benchmarking LE and deblurring networks is difficult due to the limited models in the domain. The majority of LE models are built to enhance images and are then modified to enhance videos, which often leads to poor results, as shown in [39], where the model was able to enhance light, but when the researcher attempted to apply the modified network to videos, the outputs were artefact-riddled videos. Therefore, to create benchmarks for LE models based on video enhancement, more LE models intently built for video enhancement are required.

With deblurring networks, real-world conditions are vastly unexplored (non-uniform and blind deblurring). This manifests the same issues that plagues LE models; no real benchmark exists, and no standard quantitative means of evaluating models exists, unlike with object detection algorithms, which are widely evaluated using mAP and specifically using the COCO dataset.

### 8.3. Real-Time Models

Achieving high model performance often necessitates a trade-off with inference speed, which limits the use of state-of-the-art models to offline purposes only. One of the best LE models, MBLLEN [21], has an inference time of 23 s, and DeblurGAN, a high-performing blind deblurring algorithm, has a runtime of 0.8 s. An attempt to combine these models would lead to a model which is not suitable for real-time applications. This issue is not as prevalent in action and object recognition, as these fields have received the most attention from computer vision researchers. Models such as YOLO have achieved incredible speeds, and newer versions are able to improve on speeds without sacrificing detection accuracy. As many visual data-based algorithms employ multi-dimensional CNNs, the use of separable convolutions to decrease computations, and thus inference times, is another avenue to be explored.

## 9. Conclusions

This paper has performed a comprehensive review of various popular methods and their pitfalls in the domains of light enhancement, artefact removal, action recognition and object detection, respectively. The review also looked at various datasets used in the forementioned domains and their general description. Finally, the research looked at challenges in these fields, and future directions. From the literature reviewed, one can deduce that in a world of expanding data and adapting real-world scenarios, to allow ANNs to better understand various situations, learning must shift from the time-consuming and data-intensive guided learning strategies to unsupervised, one-shot and zero-shot. This will aid in building models that are able to understand new data never seen during training, making them more versatile and suited for real-world applications. The review also highlighted that intra-datapoint variations are key for training a model more adaptable to real-world applications, as observed in the SICE_MIX dataset where not only were datapoints different from each other, but differences were observed within each datapoint, and models trained on that dataset performed better. Unlike in image processing, video processing comes with a temporal component, which in the reviewed literature was observed to be lost when networks performed video processing, and as such, artefacts would be incurred, requiring the addition of an artefact removal stage. As uncovered in the review, current light enhancement models are plagued by time-consuming data collection and processing stages (in the case of guided learning), lack of real-world data and suboptimal noise filtering capabilities. To combat this, future researchers should move away from guided learning, as finding real-world diverse paired low-light data is near impossible and the use of synthetic data limits the real-world applications of these models. It is also recommended that models should be trained with datasets of various noise levels and that denoising and enhancing be performed jointly, as this approach produced more desirable results as compared to a cascaded approach.

## Figures and Tables

**Table 1:** Both LLNet and S-LLNet are compared with non-deep learning enhancement techniques. Dark is the synthetically darkened input data. HE is histogram equalization. CLAHE is contrast-limiting adaptive histogram equalization. GA is the gamma adjustment. HE + BM3D is histogram equalization with 3D block matching. The best models were tested with 90 synthetic and 6 natural images. The best-performing model is highlighted in bold, and the number in parenthesis reflects the number of winning instances amongst the entire dataset; thus, LLNet has the best winning streak, and HE has the worst [18]. (↑) Indicates higher values are desirable. (Reprinted from [18].)

Test Items	Dark	HE	CLAHE	GA	HE + BM3D	LLNet	S-LLNet
Average PSNR (dB), synthetic (↑)	15.7902	13.7765 (0)	14.3198 (5)	15.2692 (6)	15.1653 (20)	**19.8109 (52)**	18.2248 (7)
Average SSIM, synthetic (↑)	0.4111	0.3524 (3)	0.3255 (1)	0.4345 (2)	05127 (17)	**0.6912 (65)**	0.6066 (2)
Average PSNR (dB), natural (↑)	8.2117	11.7194 (0)	9.9473 (0)	14.6664 (2)	11.9596 (1)	**15.1154 (2)**	14.4851 (1)
Average SSIM, natural (↑)	0.1616	0.2947 (0)	0.3611 (0)	0.5338 (0)	0.5437 (2)	**0.6152 (3)**	0.5467 (1)

**Table 2:** “Bird” denotes the original optimally lit and noiseless image. “Bird-D” is the darkened version of the original image, while “Bird-D + GNx” is the darkened and noisy image with Gaussian Noise of σ = x. The same is true for the remainder [18]. The best-performing model is highlighted in bold in the “average” row. (↑) Indicates higher values are desirable.(Reprinted from [18]).

PSNR (dB) (↑)/SSIM (↑)	Dark	HE	CLAHE	GA	HE+BM3D	LLNet	S-LLNet
Bird	N/A	11.22/0.63	21.55/0.90	8.93/0.66	11.27/0.69	17.61/0.84	18.35/0.85
Bird-D	12.27/0.18	11.28/0.62	15.15/0.52	29.53/0.86	11.35/0.71	20.09/0.69	15.87/0.52
Bird-D + GN18	12.56/0.14	9.25/0.09	14.63/0.11	14.10/0.11	9.98/0.13	20.17/0.66	18.59/0.54
Bird-D + GN25	12.70/0.12	9.04/0.08	13.60/0.09	13.07/0.08	9.72/0.11	21.87/0.64	22.53/0.63
Girl	N/A	18.24/0.80	17.02/0.70	11.08/0.81	18.23/0.69	18.17/0.77	14.31/0.72
Girl-D	9.50/0.50	18.27/0.80	14.36/0.66	47.32/1.00	18.26/0.69	23.61/0.76	21.21/0.72
Girl-D + GN18	9.43/0.21	16.07/0.26	12.95/0.17	17.21/0.29	19.28/0.53	19.93/0.66	21.97/0.64
Girl-D + GN25	9.39/0.15	15.33/0.19	12.09/0.12	15.37/0.20	18.50/0.39	20.08/0.60	22.60/0.59
House	N/A	13.36/0.70	18.89/0.81	10.21/0.59	13.24/0.61	11.35/0.55	10.52/0.46
House-D	12.12/0.33	12.03/0.65	16.81/0.60	28.79/0.83	11.92/0.54	21.80/0.64	18.31/0.46
House-D + GN18	12.19/0.29	10.55/0.33	15.48/0.35	14.44/0.34	11.39/0.42	21.01/0.57	19.31/0.47
House-D + GN25	12.16/0.26	10.09/0.29	14.08/0.29	13.26/0.29	10.94/037	20.68/0.54	19.84/0.47
Pepper	N/A	18.61/0.90	18.27/0.76	10.29/0.72	18.61/0.84	10.53/0.66	10.01/0.64
Pepper-D	10.45/0.37	18.45/0.85	15.46/0.61	32.97/0.90	18.45/0.80	21.52/0.79	19.2710.70
Pepper-D + GN18	0.45/0.19	14.69/0.21	14.49/0.17	15.74/0.22	16.97/0.57	22.76/0.68	22.07/0.64
Pepper-D + GN25	10.41/0.15	13.67/0.15	13.31/0.13	14.33/0.16	15.96/0.36	22.94/0.61	23.17/0.61
Town	N/A	17.55/0.79	16.35/0.69	10.02/0.76	17.70/0.76	16.28/0.80	16.03/0.78
Town- D	10.17/0.36	17.55/0.79	15.00/0.65	36.80/0.97	17.72/0.76	21.42/0.75	19.90/0.68
Town- D + GN18	10.19/0.19	14.85/0.26	13.34/0.18	15.53/0.25	17.51/0.42	19.85/0.65	20.52/0.59
Town- D+ GN25	10.21/0.14	14.22/0.20	12.40/0.13	14.08/0.17	16.62/0.32	21.63/0.60	22.89/0.58
Average	10.95/0.24	14.22/0.48	15.26/0.43	18.65/0.51	15.18/0.54	**19.66/0.67**	18.86/0.61

**Table 4:** Comparison of various zero-shot enhancers on various datasets using NIQE (↓) metric. The best-performing model is highlighted in bold. (↓) Indicates lower values are desirable. (Reprinted from [40].)

Method	DICM	ExDark	Fusion	LIME	MEF	VV	NPE	Average
ExCNet	3.6243	4.1277	3.6012	**3.7745**	**3.3595**	2.7976	4.0955	3.6258
Zero-DCE++	2.8871	4.2328	3.5168	3.971	3.3967	3.0947	**4.0231**	**3.5789**
RUAS	4.0802	4.4603	4.6601	4.2463	3.8297	4.6137	5.5342	4.4892
SGZ	**2.874**	4.237	3.4899	3.9556	3.3857	3.1413	4.0443	3.5896
ZERRINNet	3.417	**4.0199**	**3.4465**	3.8987	3.4528	**2.7879**	4.0907	3.5876

**Table 5:** Quantitative comparison of deep learning LE models which employ some form of Retinex theory. Comparison performed using NIQE (↓) metric. The best-performing model is highlighted in bold. (↓) Indicates lower values are desirable. (Reprinted from [45].)

Model	Dataset										
	LIME	LOL	DICM	VV	MEF	NPE	LSRW	SLL	ExDark	Avg	Time (s)
LIME [43]	4.109	8.129	3.86	2.494	3.576	3.658	3.655	6.372	4.588	4.542	1.635
NPE [31]	3.578	8.158	3.736	**2.471**	3.337	3.426	3.576	5.771	4.22	4.337	226.522
JieP [46]	3.719	6.872	3.678	2.765	3.39	3.522	4.015	5.622	4.215	4.260	2.606
PM-SIRE [47]	4.05	7.506	3.978	3.01	3.45	3.531	3.984	5.435	4.383	4.410	14.778
WV-SRIE [27]	3.786	7.286	3.898	2.849	3.474	3.45	3.826	5.453	4.241	4.310	56.755
MSRCR [48]	3.939	8.006	3.948	2.814	3.688	3.78	3.872	5.574	4.904	4.573	3.086
CRM [5]	**3.854**	7.686	3.801	2.617	3.264	3.562	3.721	6.008	4.525	4.391	2.840
EFF [49]	3.859	7.515	3.845	2.807	3.329	3.54	3.879	5.747	4.514	4.390	1.984
Pmea [50]	3.843	8.281	3.836	2.573	3.431	3.598	3.694	6.237	4.296	4.493	2.606
RetinexNet [35]	4.597	8.879	4.415	2.695	4.41	4.464	4.15	7.573	4.551	5.142	0.549
KinD [34]	4.763	4.709	4.15	3.026	3.876	3.557	3.543	**4.45**	4.34	3.956	2.423
RetinexDIP [51]	3.735	7.096	3.705	2.496	3.245	3.638	4.081	5.8828	4.234	4.297	61.732
RRDNet [52]	3.936	7.436	3.637	2.814	3.508	-	4.126	5.524	4.01	4.374	1021.1
KinD++ [53]	4.385	4.616	3.804	2.66	3.738	-	**3.354**	5.09	4.343	3.999	6.587
IBA [54]	4.062	7.884	3.723	3.31	3.536	3.63	3.728	5.837	4.273	4.490	0.503
Self-supervised Network [55]	4.819	3.753	4.717	3.548	4.351	4.602	4.061	5.4	4.048	4.310	**0.414**
TBEFN [44]	3.954	**3.436**	**3.503**	2.884	**3.227**	**3.292**	3.478	4.648	**3.621**	**3.511**	0.444

**Table 7:** Quantitative comparisons between DRBN and BL as reported by [70]. Best results are in bold. PSNR, SSIM and LPIPS contain paired data while DE, LOE and NIQE do not contain paired data. (Reprinted from [70].)

Metrics		DRBN	BL
MIT	PSNR	15.209	**20.13**
	SSIM	0.6684	**0.8413**
	LPIPS	0.3153	**0.1799**
	DE	6.6012	**7.252**
	LOE	678.45	**183.42**
	NIQE	5.0958	**4.0318**
LOL	PSNR	19.398	**20.427**
	SSIM	0.7223	**0.7331**
	LPIPS	0.252	**0.1305**
	DE	**6.9074**	6.6595
	LOE	619.19	**314.77**
	NIQE	45934	**4.5289**

**Table 8:** Quantitative comparison of various light enhancement techniques versus a combined approach, evaluated on the LOLv1 dataset. The best-performing model for each metric is bold. (Reprinted from [72].)

Method	PSNR	SSIM	LPIPS	FLOPs (G)	Type
RetinexNet [35]	16.77	0.419	0.474	584.5	CNN
KinD [34]	17.65	0.775	0.207	35.0	CNN
ZeroDCE [8]	14.86	0.559	0.335	4.8	Zero-shot
RUAS [64]	16.41	0.500	-	0.8	Unsupervised
LLFlow [74]	21.15	0.854	0.119	358.4	Flow
Restormer [75]	22.37	0.816	0.108	144.3	Transformer
LEDNet [76]	20.63	0.823	0.118	35.9	CNN
Retinexformer [77]	25.15	0.846	0.131	15.85	Transformer
GSAD [78]	22.77	0.852	0.102	-	Diffusion
Diff-LLE [79]	22.24	0.792	-	56.86	Diffusion
HVI-CIDNet [80]	23.50	0.870	0.086	7.57	Transformer
DiffLight [72]	25.85	0.876	0.082	168.3	Mixed

**Table 9:** (**a**) Deblurring algorithms’ dataset information. (Reprinted from [88,95]). (**b**) Deblurring algorithms’ summaries and limitations. (Reprinted from [88,95]). (**c**) Self-Reported Quantitative comparison amongst artefact removal models. (↑) Indicates higher values are desirable. (Reprinted from [88,95]).

(**a**)					
**Model**	**Deblurring Type**	**Training Datasets (Size)**	**Test Datasets (Size)**	**Videos**	**Images**
Fang et al. [84]	non-blind deblurring	BSD (200), DIV2K (800), WEP (4744)	Levin et al. (32), Sun et al. (640), Lai et al.	No	Yes
DeblurGAN [85]	Blind deblurring	GoPro, MS COCO, Unique	GoPro (1111), Kohler (4)	No	Yes
DeblurGAN-V2 [87]	Blind deblurring	GoPro, DVD, NFS	DVD, Kohler, GoPro, Lai et al., Restore	No	Yes
FFTformer [96]	Blind deblurring	GoPro, RealBlur	GoPro, RealBlur, HIDE	No	Yes
Whyte et al. [92]	non-uniform deblurring	unique	Levin et al., Joshi et al.	No	Yes
Hybrid Deblur Net [97]	non-uniform deblurring	E2VID (1000), GoPro	Real Blurry, GoPro	No	Yes
Kruse et al. [94]	uniform deblurring	Berkeley Segmentation,	Levin et al. (32), Sun et al. (640)	No	Yes
(**b**)					
Model	**Summary**		**Limitations**		
Fang et al. [84]	Introduces two error terms to prevent ringing artefacts in the deblurred image		Larger more diverse datasets produce artefact due to network		
DeblurGAN [85]	leverages Conditional Generative Adversarial Networks (cGANs) to recover sharp images from motion blurred ones		Non-real-time speeds of 0.85 s and poor performance on real-world data.		
DeblurGAN-V2 [87]	end to end GAN, based on a relativistic conditional GAN with a double-scale discriminator		Slower inference times than DeblurGAN-V1		
FFTformer [96]	Proposed Frequency domain self-atttention solver for simplified operations and gated mechanism in feed forward network		High computational resources required by transformers		
Whyte et al. [92]	Proposes using single global descriptor for blurs caused by 3D camera shake		Very long processing time and only handles blurs from 3- to 5-degree rotation		
Hybrid Deblur Net [97]	Uses event camera along with a recurrent encoder–decoder architecture to capture temporal information and remove blur noise		Requires additional equipment (event camera), deblurring only occurs at 30 fps		
Kruse et al. [94]	Regularization based on ConvNet instead of FFT-based deConv. Proposes new boundary adjustment method.		Non-real-time speeds, and model speeds heavily dependent on input image.		
(**c**)					
**MODEL**	**PSNR(↑)**		**SSIM(↑)**	**Testing Dataset**	
Fang et al. [84]	35.01		0.9426	Levin’s [98]	
Kruse et al. [94]	35.09		-	Levin’s [98]	
DeblurGAN [85]	28.7		0.958	GoPro [90]	
DeblurGAN-V2 [87]	29.55		0.934	GoPro [90]	
FFTformer [96]	34.21		0.9692	GoPro [90]	
Hybrid Deblur Net [97]	32.25		0.9285	GoPro [90]	

**Table 10:** Quantitative comparison of deblurring models. Where (**↑**) indicates higher values are desirable, the opposite is true. The best model is highlighted in red, the next best model is highlighted in blue. (Reprinted from [103].)

Method	PSNR (↑)	SSIM (↑)	LPIPS (↓)
Cascaded			
RUAS [64]→ MIMO [104]	17.81	0.569	0.523
Chen [107] → ZeroDCE	17.02	0.502	0.516
DeblurGAN-v2 → Zero DCE	18.33	0.589	0.476
MIMO → Zero DCE	17.52	0.570	0.498
Retrained and uncascaded			
SNR [106]	22.45	0.770	0.389
DeblurGAN-v2	22.30	0.745	0.356
DMPHN [108]	22.20	0.817	0.301
MIMO	22.41	0.835	0.262
Joint Light enhancement and deblurring			
LEDNet [76]	25.74	0.850	0.224
Ye [103]	26.73	0.866	0.199

**Table 11:** Quantitative survey between VJT model and deblurring models. Best model indicated in bold text. (Reprinted from [109].)

Methods	PSNR (↑)	SSIM (↑)
ESTRNN [111]	23.72	0.7600
FGST [112]	22.9434	0.6997
VRT [113]	23.37	0.7430
RVRT [114]	24.71	0.7612
VJT	**25.45**	**0.8083**

**Table 12:** Summary of other action recognition models.

Model	Pros	Cons
End-to-End	Good at capturing temporal information	Bad at capturing spatial information
Multi-stage	Good at capturing both spatial and temporal information	Long inference and training times
TURN	autonomously identifies important actions in long videos	Poor at capturing spatial relations
Temporal Context Network	autonomously identifies important actions in long videos	Poor at capturing spatial relations
Single-stream R-C3D	Captures spatiotemporal segments in videos of candidate activities	Requires large computing power
SSN	Reduces computational needs when computing normalizers	more complex than standard BN.
Two-stream R-C3D	Computation costs are shared amongst two streams	No interactions between the streams, only fusion.
BSN	Generates temporal boundaries using both local and global features	obtaining global and local features requires higher computing power
MGG UNet	Segments generated at different granularity using two branches	No branch interaction and higher computation costs.
BMN	Ensures both boundary precision and reliable confidence scores	Produces segment-wise labels instead of frame-wise

**Table 13:** YOLO versions over the years. (Reprinted from [120].)

Version	Date	Framework	Backbone
YOLO	2015	Darknet	Darknet24
YOLOv2	2016	Darknet	Darknet24
YOLOv3	2018	Darknet	Darknet53
YOLOv4	2020	Darknet	CSPDarknet53
YOLOv5	2020	Pytorch	YOLOv5CSPDarknet
PP-YOLO	2020	PaddlePaddle	ResNet50-vd
Scaled-YOLOv4	2021	Pytorch	CSPDarknet
PP-YOLOv2	2021	PaddlePaddle	ResNet101-vd
YOLOR	2021	Pytorch	CSPDarknet
YOLOX	2021	Pytorch	YOLOXCSPDarknet
PP-YOLOE	2022	PaddlePaddle	CSPRepResNet
YOLOv6	2022	Pytorch	EfficientRep
YOLOv7	2022	Pytorch	YOLOv7Backbone
DAMO-YOLO	2022	Pytorch	MAE-NAS
YOLOv8	2023	Pytorch	YOLOv8CSPDarknet
YOLO-NAS	2023	Pytorch	NAS

**Table 14:** Performance evaluation of light-enhanced detectors. The best performing model is highlighted in bold. (Reprinted from [8]).

Method	IOU Thresholds		
	0.5	0.7	0.9
input	0.231278	0.007296	0.000002
SRIE [27]	0.288193	0.012621	0.000007
LIME	0.293970	0.013417	0.000007
Li et al. [126]	0.243714	0.008616	0.000003
LightenNet [127]	0.290128	0.012581	0.000005
MBLLEN	0.289232	0.013696	0.000007
RetinexNet [35]	**0.304933**	**0.017545**	0.000005
Wang et al. [128]	0.280068	0.011107	0.000003
EnlightenGAN [59]	0.276574	0.013204	**0.000009**
Zero-DCE	0.303135	0.014772	0.000005
Zero-DCE++	0.297977	0.014587	0.000005

**Table 15:** DTCM (LE action detector) vs. vanilla detectors. Best performing model highlighted in bold. (Reprinted from [129].)

Models	Top 1 (%) Accuracy	Top 5 (%) Accuracy
3D-MobilenetV1 [130]	2.97	10.89
3D-MobilenetV2 [130]	5.93	18.04
3D-ShufflenetV1 [130]	5.49	16.48
3D-ShufflenetV2 [130]	-	-
3D-Squeezenet [130]	6.95	19.53
3D-ResNet-18 [131]	18.25	36.28
3D-ResNet.50 [131]	20.16	36.94
3D-ResNet-101 [131]	21.78	38.74
3D-ResNext-101 [131]	22.30	42.27
GT-I3D [132]	23.24	-
DTCM	**27.43**	**45.28**

**Table 16:** LE popular datasets.

Dataset	Size	Paired	Real	Synthetic	Video	Image	Resolution and FPS
SDSD [133]	37,500	Yes	Real	No	Yes	Yes	Mixed
LOL [35]	500	Yes	Yes	No	No	Yes	400 × 600
SID [134]	5094	Yes	Yes	No	No	Yes	4240 × 2832; 6000 × 4000
SICE	4800	No	Yes	Yes	No	Yes	Mixed
SMOID [135]	179	Yes	Yes	No	Yes	No	1800 × 1000
SICE_Mix	589	Yes	Yes	Yes	No	Yes	600 × 900
MIT-Adobe FiveK [136]	5000	No	Yes	Yes	No	Yes	Mix

**Table 17:** Artefact removal datasets.

Dataset	Size	Pair	Real	Syn.	Video	Image	Type	Res and FPS	Domain
RBI [137]	55	Yes	Yes	No	Yes	No	Mixed	25 and 500 fps	Scene
DVD [138]	6708	Yes	No	Yes	Yes	No	Uniform	30 and 240 fps	Mixed
Shen et al. [139]	120 M+	Yes	No	Yes	No	Yes	Uniform	Mixed	Face
REDS	300	Yes	No	Yes	Yes	No	Uniform	Mixed	Scene
HIDE	8422	Yes	No	Yes	No	Yes	Uniform	-	People and Objects
GoPro	3214	Yes	No	Yes	No	Yes	Uniform	1280 × 720	Scene

**Table 18:** Criminal actions and weapons datasets.

Dataset	Size	Type	Real	Syn.	Video	Image	Weapons	Res. and FPS
UCF-Crime	1900	Mixed	Yes	No	Yes	No	Yes	Mixed
Bianculli et al. [140]	350	Mixed	No	Yes	Yes	No	Yes	1920 × 1080
RWF-2000 [141]	2000	Contact	Yes	No	Yes	No	No	30 fps
VSD [142]	1317	Mixed	No	Yes	Yes	No	Yes	Mixed
Qi et al. [143]	51 k+	Guns	Yes	No	No	Yes	Yes	Mixed
CCTV-Fights [144]	1 K+	Contact	Yes	No	Yes	No	No	Mixed
Castillo et al. [145]	10 k+	Mixed	Yes	Yes	Yes	Yes	Yes	Mixed

## Data Availability

The data presented in this review are available as follows; for low-light enhancement https://github.com/ShenZheng2000/LLIE_Survey, accessed on 10 June 2024; for artefact removal, https://github.com/subeeshvasu/Awesome-Deblurring, accessed on 22 September 2024 and for recognition and detection, https://github.com/jinwchoi/awesome-action-recognition, accessed on 6 December 2024.
